# Statistik zwischen Data Science, Artificial Intelligence und Big Data: Beiträge aus dem Kolloquium „Make Statistics great again“

**DOI:** 10.1007/s11943-022-00305-7

**Published:** 2022-05-19

**Authors:** Ulrich Rendtel, Willi Seidel, Christine Müller, Florian Meinfelder, Joachim Wagner, Jürgen Chlumsky, Markus Zwick

**Affiliations:** Fachbereich Wirtschaftswissenschaft, Institut für Statistik und Ökonometrie, Freie Universität Berlin, Berlin, Deutschland

**Keywords:** Statistik Studiengänge, Survey Statistk, Amtliche Statistik, Datenzugang, Data Science, Statistical Literacy, Big Data, Künstliche Intelligenz, Informatik, Programmes in statistics, Survey statistics, Official statistics, Data access, Data science, Statistical literacy, Big data, Artificial intelligence, Informatics, A12, C18, C45

## Abstract

Die Statistik als fachliche Disziplin muss sich in einem rasch wandelnden Umfeld behaupten, das durch den Aufstieg der Data Science, den Bedeutungszuwachs der künstlichen Intelligenz sowie neue Datenstrukturen charakterisiert wird. Wie kann sich die Statistik hier behaupten oder verlorenes Terrain wiedergewinnen? Unter dem provokanten Motto „Make Statistics great again“ wurden aus verschiedenen Blickwinkeln Entwicklungen, Strategien und positive Beispiele skizziert, wie sich das Fach Statistik an der Universität, im Wissenschaftsbetrieb und auf dem Arbeitsmarkt positionieren sollte. Willi Seidel schaut aus dem Blickwinkel eines Hochschulpräsidenten auf den Ressourcenkampf der Fächer. Christine Müller berichtet über die Initiativen der Dachorganisation DAGStat die vielen Teildisziplinen der Statistik wirkungsvoll im Wissenschaftsbetrieb und in der Öffentlichkeit zu positionieren. Florian Meinfelder dokumentiert den Aufstieg des Masterstudiengangs in Survey Statistik zu einem der nachgefragtesten Studiengänge der Uni Bamberg. Jürgen Chlumsky und Markus Zwick beleuchten die historische Wahrnehmung der Amtsstatistik bei Pflichterhebungen und die Entwicklung der Forschungsdatenzentren sowie moderner Zugänge zu neuen Datenquellen. Joachim Wagner schildert das Verhältnis von Datenproduzenten und Datennutzern aus der Sicht eines unzufriedenen Datennutzers. Schließlich geht es um die Position der Statistik in der Datenwissenschaft. Ist „Data Science“ nur ein neumodisches Wort für Statistik? Ein Konzeptionspapier der Gesellschaft für Informatik (GI) hat zu Positionspapieren der DStatG und der DAGStat geführt, die von Ulrich Rendtel vorgestellt werden.

Das Kolloquium fand anlässlich der Abschiedsvorlesung von Ulrich Rendtel im Juni 2019 am Fachbereich Wirtschaftswissenschaft der Freien Universität statt.

## Abstract (en)

Statistics as a subject has to sustain its position in a fast changing environment which is characterized by the rise of Data Science, the growth of the importance of Artificial Intelligence and the emergence of new Data Structures. How can Statistics keep its position or even regain lost territory? Under the provoking motto “Make Statistics great again” we sketch under different views developments, strategies and positive examples, how the subject statistics can evolve at universities, in scientific competition and at the labor market. Willi Seidel comments the competition of subjects for resources from the viewpoint of the head of a university. Christine Müller reports the initiatives of the DAGStat umbrella organization, to promote the different subbranches of statistics in the scientific competition and in the public view. Florian Meinfelder documents the rise of the master program of Survey Statistics to one of the most requested master programs at the university of Bamberg. Jürgen Chlumsky and Markus Zwick report the public notion of mandatory surveys of official statistics as well as the development of research data centers and alternative access to new data sources. Joachim Wagner comments the relationship of data producers and data users from the view of a discontent data analyst. Finally, the position of statistics in the subject data science is discussed. Is the term “Data Science” only a modern phrase for statistics? A White Paper of the Gesellschaft für Informatik (GI) has provoked two position papers of the DStatG and the DAGStat which are presented by Ulrich Rendtel.

The colloquium took place at the farewell lecture of Ulrich Rendtel at the Economic Faculty of the Freie Universität Berlin in June 2019.

## Einleitung

*Ulrich Rendtel*

Der Ruf der Statistik ist nicht immer positiv. Statistische Schwankungen werden häufig als Fehler bezeichnet, die mit einer unsicheren oder undurchsichtigen Methodik in Verbindung gebracht werden. Landläufig hält man statistische Zusammenhänge für obskur, unzuverlässig, zweifelhaft. Der Volksmund spricht auch schon mal von vorsätzlichen Lügen. Die Vertreter dieses Fachs gelten häufig als unverständlich dreinredende Nerds, die ihren Formelapparat mehr lieben als den Kontakt zur Wirklichkeit. Wenn es die Zunft der Statistiker mal geschafft hat, sehr freundlich beurteilt zu werden (Hal Varian, der Chefökonom von Google, sagte in einem Interview im Oktober 2008: I keep saying the sexiest job in the next ten years will be statisticians (https://flowingdata.com/2009/02/25/googles-chief-economist-hal-varian-on-statistics-and-data/ Abruf 30.09.2020)), wird die Nennung der Statistik gleich durch alternative Begriffe getilgt. Gerne wird Statistik durch Data Science ersetzt. So loben Davenport und Patil (2012) den Data Scientist gleich als „The Sexiest Job of the 21st Century“ aus. Auch bei der Fähigkeit, Zahlen zu interpretieren, also der Literacy, hat die „Data Literacy“ die Führung vor der „Statistical Literacy“, vgl. die Diskussion und Abgrenzung dieser Begriffe in Krämer et al. (2019) sowie Schüller (2019). Eine Vorlesung mit dem Titel „Multivariate Verfahren“ ist out, dafür gibt es „Predictive Analysis“, „Business Intelligence“, „Künstliche Intelligenz“. Lange war auch das Modewort „Data Mining“ in. Alles scheint erlaubt, aber bitte nicht ein Titel aus dem klassischen Arsenal der Statistik. Solche Klagen werden immer wieder angestimmt. Viele Fachvertreter sehen sich als Anbieter des „least liked subjects“ vom Aussterben bedroht^1^.

Es gibt allerdings auch Gegenstimmen, die diese pessimistische Sicht nicht teilen und ganz hoffungsvoll „The Future of Statistics“ beschreiben, vgl Rendtel (2012), Egeler et al. (2012) oder Radermacher (2012). Wo verläuft der Weg zu einer Aufwertung des Fachs Statistik? Was sind seine Stationen? Wo wurde er erfolgreich beschritten? Dieses war das Thema eines Kolloquiums unter dem Titel „Make Statistics great again“^2^. Das Kolloquium fand anlässlich der Abschiedsvorlesung von Ulrich Rendtel im Juni 2019 am Fachbereich Wirtschaftswissenschaft der Freien Universität statt.

Einige der Referenten haben ihr teilweise sehr persönliches Statement jetzt schriftlich nachgereicht^3^. Ihr Zusammenhang wird im hier folgenden etwas ausführlicheren Editorial hergestellt.

### Ein ehemaliger Kollege als Leiter einer Universität

Ein Kanal zu Einfluss, Anerkennung, Stellenzuwachs und Ausstattung läuft über die Hochschulleitung. Hier gab es den seltenen Fall, dass ein Statistiker die Leitung einer Hochschule übernommen hat. Willi Seidel war lange Jahre an der Helmut-Schmidt-Universität der Bundeswehr in Hamburg Statistik-Professor, bis er 2010 die Leitung der Universität übernahm, die er bis 2018 innehatte. Nebenher war er bis 2012 Vorsitzender der Deutschen Statistischen Gesellschaft. Unter seiner Ägide wurde die Helmut-Schmidt-Universität stark ausgebaut. Es wurden neue Studiengänge eingerichtet und der Stellenkegel wurde um fast 30 % vergrößert. Ist die Statistik hier größer geworden? Wie hat der ehemalige Statistiker sein Fach in Konkurrenz mit anderen Studiengängen wahrgenommen? Und gab es in Hamburg, einer Stadt mit mehreren Universitäten, einem berühmten Universitätsklinikum und diversen Forschungseinrichtungen eine übergreifende Aktivität von Statistik-Treibenden?

### Statistik unter einem Dach

Ökonometrie, Biometrie, Empirische Sozialforschung, Epidemiologie … Die Liste der Fächer, die statistische Methoden benutzen, ist lang. Die Statistik als methodologische Klammer ist ein Querschnittsfach per se! Was bringt es, wenn man diese einigende Klammer auch organisatorisch stärkt? Dieser Versuch läuft seit 2005 und heißt Deutsche Arbeitsgemeinschaft Statistik (DAGStat). Christine Müller war sechs Jahre die Vorsitzende der DAGStat. Sie berichtet über die zähen Bestrebungen, die Statistik als Fach voranzubringen.

### Das Amt: Eine feste Burg der Statistik?

Älter als die vielen „Metrien“ ist die amtliche Statistik. Und diese führt den Produktnamen „Statistik“ ohne allen modischen Schnickschnack auf ihrem Briefkopf. Die amtliche Statistik betrachtet sich als die logistische Basis für eine evidenzgeleitete Gesellschaftspolitik. Der Grundstock besteht aus Erhebungen, die per Gesetz angeordnet werden^4^. Dies impliziert eine starke Stellung der Juristen im amtlichen Statistikbetrieb^5^. Auch im Amt ist der Hunger nach „Big Data“ und neuen Auswertungsmethoden gestiegen. Selbstbewusst konkurriert man auf dem Ausbildungs- und Absolventenmarkt unter dem Label „European Master in Official Statistics (EMOS)“ um Universitätsabgänger. Markus Zwick kennt beide Seiten: Das Amt und die Universität und vor allem auch die europäische Perspektive. Jürgen Chlumsky hat lange Zeit das Institut für Forschung und Entwicklung im Bundesamt geleitet. Sein Anliegen ist die Politikberatung durch die Statistik, die sich beispielsweise in den Grohmann-Vorlesungen auf der Statistischen Woche manifestiert, vgl. Chlumsky (2012).

### Datenzugang und Datenschutz

Daten sind der Stoff, aus dem Statistiker verwertbare Erkenntnisse und Einsichten produzieren. Ohne Zugang zu Daten ist der Statistiker aufgeworfen. Hier wurde mit der Einrichtung der Forschungsdatenzentren und eines Rats für Sozial- und Wirtschaftsdaten (RatSWD)^6^ ein großer Fortschritt erreicht.

Es gibt jedoch an mehreren Stellen Reibungspunkte zwischen den Datenproduzenten und den Datennutzern: Was wird überhaupt erfragt bzw. erhoben? Und zu welchen Konditionen kommt man wann an die erhobenen Daten heran? Dies ist nicht nur eine Frage des Preises, sondern auch des Datenschutzes. Das Bundesverfassungsgericht hat in seinem Urteil zur Volkszählung der Wissenschaft ein Privileg zugestanden: den Zugang zu faktisch anonymisierten Daten, die deswegen auch als sogenannte „Scientific Use Files“ bezeichnet werden. Doch hinter diesem schönen Namen steckt ein potenzielles Betrugsszenario. Man bezeichnet einen Datensatz als faktisch anonymisiert, wenn der Aufwand zu einer Deanonymisierung, die verboten ist, unverhältnismäßig groß ist, so dass derartige Aktivitäten sich nicht lohnen. Es ist in seinem Kern ein Worst-Case Szenario. Der Datenlieferant verhält sich so, als ob der Datennutzer sich nicht an die Regeln hält. Die Analysemöglichkeiten unter einem alternativen Paradigma, das auf Vertrauen baut, warten in Deutschland noch auf ihreRealisierung, vgl. Rendtel (2014).

Joachim Wagner war unter den Ersten, die Firmendaten im Längsschnitt analysiert haben. Er ging damit den immer wiederkehrenden Meldungen über die wohltuenden Aktivitäten des Mittelstands am Arbeitsmarkt empirisch nach. Viele der im Wirtschaftsteil angesehener Zeitungen behaupteten Zusammenhänge lassen sich nach seiner Einschätzung empirisch nicht belegen, vgl. Wagner (2015). Als Mitglied auf der Wissenschaftler-Bank des RatSWD hat er die Interessen der Wissenschaftler gegenüber den Datenproduzenten vertreten. Sein Plädoyer für einen verbesserten Datenzugang würde den Statistikern genauere Analysen ermöglichen und so im Kampf um die öffentliche Wahrnehmung mehr Aufmerksamkeit geben.

### Eine Erfolgsgeschichte: Der Master in Survey Statistik

Die Erhebung von Daten und die Durchführung von Umfragen gehören zu den Kernbereichen der Statistik. Hinzu kommen neue Möglichkeiten des Datenzugangs über das Internet, über Mobilfunk und Bewegungsdaten oder über Register, vgl. Zwick (2016). Auch die Verknüpfung dieser unterschiedlichen Datenquellen (Schmid et al. 2017) erweitert die Möglichkeiten zur Auswertung klassischer Surveys. Also ein lohnendes Feld für einen spezifischen Studiengang! Aber wie kriegt man so etwas realisiert? Es gibt in Deutschland nur eine Handvoll Lehrstühle, die sich mit Survey Statistik beschäftigen. Die Antwort war eine Kooperationslösung, also der Austausch von Lehrveranstaltungen via Videokonferenz. Begonnen hatte dieses Experiment 2010, als Webex und Zoom noch nicht existierten und man für alle Teilnehmer einen speziellen Raum mit Equipment für die Durchführung der Videokonferenz brauchte. Es war der persönliche Einsatz von drei Kollegen (Susanne Rässler, Ralf Münnich und Ulrich Rendtel) aus drei verschiedenen Bundesländern, die alle administrativen Hürden ignorierten und einen Master in Survey Statistik^7^ in Bamberg, Trier und Berlin starteten. Florian Meinfelder berichtet vom Aufstieg des Master Survey Statistik zu einem der beliebtesten Masterprogramme seiner Universität.

Einigkeit macht also stark. Auf dem Kolloquium berichtete Markus Zwick über ein weitaus größeres Kooperationsprojekt, das von Eurostat unterstützt wird und die Ausbildung fähiger und innovativer Statistiker in der amtlichen Statistik (Official Statistics) zum Ziel hat: Der European Master in Official Statistics (EMOS)^8^. Die Entstehung, Ziele und Absichten von EMOS hat Zwick (2015) beschrieben. Mittlerweile nehmen 32 Master Programme aus 19 Ländern am EMOS Programm teil. Notwendige Bestandteile des Programms sind ein Praktikum sowie eine Masterarbeit im Bereich der amtlichen Statistik. Hier steht EMOS jedoch in harter Konkurrenz zu Bereichen, die ihre Praktika besser vergüten. Immerhin hat eine Berliner Statistik-Studentin mit ihrer Masterarbeit zwei akademische Preise^9^ und einen Arbeitsplatz beim Amt für Statistik Berlin/Brandenburg gewonnen.

### Das Verhältnis zur Informatik: Oder wer hat das Sagen im Datenwald?

Ein aktueller Hype ist Data Science. Die deutsche Bezeichnung als Datenwissenschaft wird eher selten benutzt. Zur Zeit sprießen unter diesem Namen neue Studienprogramme nur so aus dem Boden; an der LMU in München sogar als Elitestudiengang^10^. Doch auch bestehende Studiengänge wie der Bachelor Statistik der LMU firmiert jetzt als „Statistik und Data Science“ (https://www.lmu.de/de/studium/studienangebot/alle-studienfaecher-und-studiengaenge/statistik-und-data-science-bachelor-hauptfach-3019.html). Weitere Statistik-affine Ausbildungsziele in Zeiten von Big Data präsentiert Zwick (2016). Auch wenn man sich als Statistik-Professor zur Zeit darüber freuen kann, dass unsere Absolventen am Arbeitsmarkt so gute Chancen wie noch nie haben, so liegt dies vielleicht nur an einem eklatanten Mangel an ausgebildeten Data Scientists. So etwa lautet die Warnung einer Expertengruppe der National Science Foundation (NSF) der USA zur Statistik am Scheideweg (Statistics at Crossroads. vgl. He et al. (2019)).

Aber ist die Statistik nicht die Wissenschaft der Realisierung von Beobachtungen und der Auswertung von Daten? Ist Data Science damit nichts anderes als ein Synonym für Statistik? Offensichtlich wird das anderswo anders gesehen. Historisch gesehen geht der Begriff auf Cleveland (2001) zurück, der eine Abkehr von der Mathematisierung der Statistik forderte und die stärkere Betonung der Anwendungsseite.

Dieser Botschaft ist auch die Statistik-Professur an der FU mit der Einrichtung der statistischen Beratungseinheit fu:stat^11^ nach dem Vorbild des Statistischen Beratungslabors Stablab^12^ an der LMU gefolgt. Dessen Leiter, Helmut Küchenhoff berichtete auf dem Kolloquium über das Verhältnis von Statistik und Informatik unter dem Titel „Künstliche Intelligenz, Machine Learning, Big Data, Business Analytics, Data Science, alles Statistik!?“ Ganz so eindeutig war sein Resümee nicht. Man könne und solle auch von den Informatikern lernen. Und ein bisschen auf die Modewörter einzugehen, kann auch nicht schaden. Aber man sollte den Machine-Learnern auf den statistischen Zahn fühlen! In ihrem Aufsatz „Statistik, Data Science und Big Data“ haben Kauermann und Küchenhoff (2016) dieses Verhältnis näher beschrieben und anhand von Beratungsfällen dargestellt. Kauermann und Seidl (2019) beschreiben Data Science aus der Sicht eines Statistikers und erkennen an, dass umgekehrt Statistiker von den Informatikern noch einiges lernen können.

Aber wie sieht die Datenwissenschaft aus der Sicht eines Informatikers aus? Hier löste im Nachgang des Kolloquiums ein White-Paper der Gesellschaft für Informatik (GI) eine heftige Diskussion aus, die in zwei programmatischen Stellungnahmen der DStatG und der DAGStat mündete. Das GI-Papier und die beiden Stellungnahmen ergänzen diesen Programmpunkt des Kolloquiums. Sie sollen daher am Schluss dieses Hefts behandelt werden.

### Make Statistics great again?

Täuschen wir uns nicht: Unser Fach ist zwar großartig, aber über viele Fächer und Institute verstreut und schon gar nicht mächtig. Mögliche Strategien, dies ein wenig zu ändern, liegen in der Kooperation mit unseren fachlichen Kollegen, ob in derselben Stadt oder in verschiedenen Bundesländern, die Studiengänge ermöglicht, in denen Statistik das Hauptfach ist. Die Sammlung des Querschnittsfachs Statistik unter einem organisatorischen Dach vermindert die Diaspora, fördert den Austausch und macht uns sichtbarer. Und wir müssen uns den Anwendern noch stärker öffnen und vielleicht auch die Scheu vor Modewörtern überwinden. Dabei können wir selbstbewusst auf den Kern unserer Wissenschaft zurückgreifen: wie man Daten erhebt und wie man interpretierbare Analyseergebnisse gewinnt. Damit werden wir vielleicht nicht „Great“ werden aber vielleicht doch etwas größer.

## Wie sieht die Statistik aus der Perspektive eines Hochschulleiters aus?

*Willi Seidel*

Kollege Rendtel hatte mich eingeladen, im Rahmen seines Abschiedskolloquiums die Sicht eines Hochschulleiters auf das Fach Statistik zu schildern. Tatsächlich war ich bis März 2018 Präsident der Helmut-Schmidt-Universität/Universität der Bundeswehr Hamburg (HSU), sollte das also können. Hintergrund war die bisweilen sehr prekäre Lage „der Statistik“ an deutschen Universitäten und die Frage, ob und wie man Hochschulleitungen motivieren könne, dieser entgegenzuwirken. Die folgenden Ausführungen schildern persönliche Eindrücke, dies ist also kein wissenschaftlicher Artikel. Ferner beschränke ich mich auf die Situation in Deutschland.

Hier also die Sicht einer idealtypischen Hochschulleiter*in:

Die Statistik ist brillant, dynamisch, cool, mit geistigem Tiefgang, kurz: Great.

Warum?

- Wenn die Hochschulleiterin Kollegin Gather ist, vormalige Rektorin der TU Dortmund, ist die Antwort definitiv korrekt. Jedenfalls gehe ich davon aus.
- Handelt es sich um die Leitung irgendeiner Hamburger Hochschule zur Zeit meiner Präsidentschaft, so stimmt die Aussage auch: Für diese Leitung war die Statistik als Fach völlig unsichtbar, also war die Voraussetzung der Frage nicht erfüllt und in etwas freier Auslegung von „ex falso quodlibet“ kann ich alles behaupten, also eben: Statistik ist great.

Zur Illustration noch die Aussage eines Amtskollegen: „Natürlich haben wir Statistik“, aber er meinte Statistiken über Studierendenzahlen, Finanzen usw., mit denen er sich laufend beschäftigte. Das Fach Statistik taucht übrigens nicht einmal in einer MINT-Studie des Wissenschaftsrates über Hamburger Hochschulen explizit auf, wohl aber zum Beispiel Bekleidung, Körperpflege und natürlich ganz viel Informatik.

Das müsste einen nicht unbedingt stören. Die Funktionalanalysis beklagt sich auch nicht darüber, dass der Präsident der Universität Hamburg (UHH) sie nicht kennt; sie ist eben essentieller Bestandteil des Fachs Mathematik, und dieses Fach ist ohne Zweifel wichtig.

Der Unterschied zur Statistik ist allerdings augenfällig. Obwohl die Statistik eine eigenständige Disziplin von erheblicher gesellschaftlicher Bedeutung ist, mit eigenem Methodenkanon und breit aufgefächerten Teilgebieten, taucht sie in Deutschland häufig nur als Unterstützungsfach anderer Disziplinen auf, wie Wirtschaftswissenschaften, Medizin, Politikwissenschaften …

Daraus resultiert ihre häufig prekäre Existenz. Sie wird nicht als eigenes (vollwertiges) Fach wahrgenommen, und organisatorisch können die für andere Disziplinen erforderlichen statistischen Kenntnisse nötigenfalls angeblich auf vielerlei andere Weisen vermittelt werden als durch eigene Professuren. Insbesondere liegt das Fach außerhalb des Wahrnehmungshorizonts vieler Hochschulleitungen, diese fallen somit als Unterstützer erst einmal aus.

### Ziel und Handlungsfelder

Das Fach Statistik muss als eigenständiges Fach von großer gesellschaftlicher Bedeutung wahrgenommen werden. Dazu braucht es:

- qualifizierte Fachvertreterinnen und Fachvertreter
- Forschungsmöglichkeiten
- Ausreichend Stellen sowie die nötigen Handlungsmöglichkeiten und Ressourcen
- Sichtbarkeit und Attraktivität für den Nachwuchs mit Qualifikations- und persönlichen Entwicklungsmöglichkeiten über alle Stufen hinweg

Falls die Statistik nicht Hauptfach ist, ist keiner dieser Punkte selbstverständlich. Sie kämpft dann beispielsweise häufig um ihren Platz in Studien‑, Prüfungs‑, Promotions- und Habilitationsordnungen und damit um Qualifikationswege und um das Interesse der Studierenden. Wenn die nicht gegeben sind, bleibt der Nachwuchs weg.

Selbstverständlich wären viele gut ausgestattete eigene Fakultäten für Statistik mit eigenen Studiengängen optimal. Wünschen darf man, aber Magie können nur wenige (TU Dortmund, LMU München).

Man kann stattdessen nach intelligenten Wegen suchen, die Statistik trotz organisatorischer Zersplitterung und Vereinzelung als eigenständiges Fach mit eigenständigem Nutzen zu präsentieren und beispielsweise adäquate Studien- und Qualifikationsangebote bereitzustellen. Eine mögliche organisatorische Lösung liegt offensichtlich in bereichsübergreifender Zusammenarbeit. Einige Anmerkungen dazu folgen unten.

Die Statistik muss auch endlich die Aufmerksamkeit erhalten, die ihr zukommt; innerhalb von Hochschulen, in der Öffentlichkeit, und bei Entscheidungsträgern in Politik und Wirtschaft, von denen letztlich die Bereitstellung von Ressourcen und von Arbeitsplätzen abhängt. Daher ist aus meiner Sicht eine breit angelegte, koordinierte Imagekampagne für die Statistik wichtig, verbunden mit einer professionellen und intensiven Lobbyarbeit. Auch dazu folgen unten Anmerkungen.

### Bereichsübergreifende Zusammenarbeit

Handlungsfähigkeit erreicht man durch Zusammenschluss und gemeinsame Vorhaben; Vielfalt der Beteiligten resultiert in höherer Qualität des Angebots, Größe in Sichtbarkeit. Zwei sich nicht gegenseitig ausschließende Modelle sind:

1. Statistiker*innen schließen sich über Fakultäts‑, Universitäts- und Institutionengrenzen hinweg zusammen; nicht im Sinn einer Fachgesellschaft, sondern um gemeinsame Forschungs- oder sonstige Vorhaben zu initiieren (auch Beratung), um gemeinsame Förderanträge zu stellen, und/oder um gemeinsame Studiengänge zu betreiben.
2. Statistiker*innen schließen sich mit Vertretern anderer Wissenschaften zusammen, um zusammen Projekte zu betreiben, in denen die Statistik eine große Rolle spielt.

Große Städte oder Metropolregionen mit zahlreichen wissenschaftlichen Institutionen sind dabei im Vorteil, andererseits sind digitalen Kommunikationstechniken keine geographischen Grenzen gesetzt. Überregional werden wohl eher thematisch homogene Verbünde entstehen. Bei gemeinsamen Studienangeboten können Ländergrenzen allerdings durchaus Hindernisse sein.

Alle diese Wege werden bereits gegangen. Zur Illustration einige Beispiele:

Zu (1): In Hamburg beispielsweise, wo die Statistik, wie beschrieben, bisher eher unsichtbar war, haben im Herbst 2019 Wissenschaftler der HSU um Kollegen Gertheiss die Initiative ergriffen und recherchiert, wer sich an Hamburger Universitäten mit Statistik beschäftigt. Darauf luden sie auf zunächst professoraler Ebene zu einem „Hamburger Statistik Workshop“ am 08. November 2019 an die HSU ein. In der Folge engagierten sich auch Wissenschaftler*innen der Universität Hamburg und des Universitätsklinikums Hamburg-Eppendorf (UKE) sehr stark; am 27. Februar 2020 fand in erheblich größerem Rahmen unter Beteiligung des wissenschaftlichen Nachwuchses am UKE ein Treffen „Statistics in Hamburg – 1st Workshop“ statt. Die Arbeitsgruppen stellten sich vor, und es wurde eine Verstetigung der Arbeit vereinbart, mit dem Ziel beispielsweise gemeinsamer Forschungsprojekte und Anträge. Teilnehmer*innen sollen überrascht gewesen sein, wieviel Statistik in Hamburg getrieben wird!

Vorbilder für die Hamburger Initiative existieren bereits an anderen Orten. Als Beispiel für eine umfassende fakultätsübergreifende Kooperation sei das Zentrum für Statistik an der Georg-August-Universität Göttingen genannt, mit einem internationalen Promotionsprogramm „Angewandte Statistik und Empirische Methoden“, einem Master „Angewandte Statistik“ und umfassendem Beratungsangebot, auch für Industrie, Handel und Verwaltung.

Andere Kooperationen konzentrieren sich auf gemeinsame Studiengänge, so bieten beispielsweise in Berlin die Freie Universität (FU), die Humboldt- und die Technische Universität sowie die Charité-Universitätsmedizin gemeinsam einen „Master of Science in Statistics“ an. Ein länderübergreifender Master in Survey-Statistik wird von den Universitäten Bamberg, Trier und FU Berlin in gemeinschaftlicher Zusammenarbeit angeboten, dieser zählt zu dem Programm „European Master in Official Statistics“ (EMOS) des Statistischen Amtes der Europäischen Union (Eurostat).

Der Sprung über Institutionengrenzen fällt leichter, je weniger man darauf angewiesen ist, dass in allen Veranstaltungen alle Studierenden an einem Ort physisch präsent sein müssen. Je nach Prüfungsordnungen können auch verschiedene Bundesländer beteiligt sein. Auch Graduiertenprogramme sind so denkbar.

Zu (2): Aufgrund der vielfältigen Einsatzfähigkeit der Statistik ist diese Variante selbsterklärend. Hier nur der Hinweis, dass man auf diese Weise beispielsweise auch dann Vertiefungsmöglichkeiten in Statistik für interessierte Studierende schaffen kann, wenn diese etwa im Rahmen eines BWL-Studiums eigentlich nicht vorgesehen sind. Ich kenne einen BWL-Masterschwerpunkt „Risikomanagement“, der im Wesentlichen von Juristen und Statistikern betrieben wird.

In beiden Varianten können aus den Zusammenschlüssen feste, auch institutionenübergreifende anerkannte Strukturen entstehen, mit dem Ziel der Grundlagen- oder angewandten Forschung oder der Dienstleistung. Ein Hamburger Beispiel aus anderen Wissenschaften ist das „Zentrum für Hochleistungsmaterialien“, eine hoch angesehene Einrichtung, die aus einem entsprechenden SFB hervorgegangen ist. Sie ist institutionenübergreifend, beteiligt sind die Technische Universität Hamburg, die Universität Hamburg, die HSU, das Helmholtz-Zentrum Geesthacht und das DESY. Weitere Beispiele gibt es aus Logistik und Luftfahrt.

### Imagekampagne und Lobbyarbeit

Bescheidenheit ist bekanntlich eine Zier, doch ebenso bekanntlich „kommt man weiter ohne ihr“. Besonders, wenn man gegen ein Fach wie Informatik bestehen muss. Ein Fach, das die sogenannte „Künstlichen Intelligenz“ vereinnahmt hat, und mittlerweile zu allem Überfluss auch noch dabei ist, Data Science zu vereinnahmen. Statistische Methoden gelten als starr und beschränkt, weit weniger leistungsfähig als KI. Obendrein gilt KI als umfassende Problemlöserin und Innovationstreiberin. Um wirtschaftlich wettbewerbsfähig zu sein, muss in KI, sprich Informatik, umfassend investiert werden.

Dieser Verdrängungswettbewerb ist skurril, wenn man bedenkt, dass Kernmethoden der KI aus der Statistik kommen. Aber er ist real. Dies ist andererseits kein Plädoyer dafür, Statistik in Abgrenzung zu/als bessere Variante von KI zu positionieren; vielmehr ist es ein Plädoyer dafür, in der Außendarstellung ebenso professionell vorzugehen.

Es braucht eine klare, verständliche Story, die einleuchtend den Nutzen der Statistik darstellt und die besonderen Merkmale der Statistik, auf denen dieser Nutzen beruht. Der Nutzen wäre zum Beispiel die Bereitstellung verlässlicher Informationen für schwierige politische, gesellschaftliche oder geschäftliche Entscheidungen von großer Tragweite. Grundlage für die Verlässlichkeit dieser Informationen sind ein methodisch abgesichertes Studiendesign sowie die Möglichkeit, Vorgänge zu verstehen, zu erklären (Interpretierbarkeit) und die Ergebnisse abzusichern (Quantifizierung der Unsicherheit).

Es braucht konkrete Beispiele, insbesondere Erfolgsgeschichten. Eigentlich könnte die Corona-Krise ein gutes Beispiel dafür sein, welchen Nutzen die Statistik für politische Entscheidungen immenser Tragweite bringen kann^13^. Nur die Statistik kann verlässliche Daten bereitstellen, durch ein korrektes Studiendesign, transparente Modelle, ausgefeilte Schätzmethoden und valide Konfidenzintervalle. Fragt sich nur, ob nicht die Statistik damit dem allgemein grassierenden Glaubwürdigkeitsverlust der Wissenschaft zum Opfer fiele.

„Data Science“ ist in aller Munde, diesen Boom sollte die Statistik nutzen, nicht nur in der Darstellung, auch in der raschen Bereitstellung entsprechender Studien- oder Ausbildungsangebote. Verbände und Banken verlangen von Unternehmen vermehrte Einstellung von Data Scientists – liefern wir ihnen diese! Es gibt weniges, was eine Hochschulleitung mehr beeindruckt, als ein erfolgreicher Studiengang.

Die Statistik sollte sich nicht scheuen, die Techniken erfolgreicher Öffentlichkeits- und Lobbyarbeit anzuwenden. Die Organisation entsprechender Kampagnen könnte die Aufgabe der DStatG oder der DAGStat sein. Ein ausreichender Etat sollte (eingeworben und) für professionellen Rat bereitgestellt werden.

Ein schönes Beispiel für eine gelungene Kommunikation über Statistik ist mir in guter Erinnerung geblieben:

Bei meiner Verabschiedung als Präsident der HSU hielt Kollegin Christine Müller den Festvortrag. Das Publikum war hochrangig, unter anderem war die damalige Bundesministerin der Verteidigung, Frau Dr. von der Leyen, anwesend. Darüber hinaus eine große Zahl von Entscheidungsträgern auch aus nichtwissenschaftlichen Bereichen. Das Thema von Kollegin Müller war Bayes’sche Statistik und ihre Anwendung in lernenden Algorithmen, beispielsweise im Smartphone. Sie verstand es, die Grundidee der Bayes’schen Statistik sehr anschaulich zu vermitteln. Ich traf in den folgenden Wochen etliche Personen aus der Hamburger Gesellschaft, die mir von dem Vortrag vorschwärmten. Sie hatten eine völlig neue Sicht auf Statistik bekommen und hatten sich nicht vorstellen können, dass Statistik in so wichtigen alltäglichen Anwendungsfeldern eine Rolle spielt. Ich konnte mir natürlich den Hinweis nicht verkneifen, dass KI eigentlich Statistik sei.

### Abschließende Bemerkungen

Erstens ist mir bewusst, dass dieser Beitrag „nur“ organisatorische und Wahrnehmungsfragen adressiert und keine fachspezifische inhaltliche Diskussion der vom Kollegen Rendtel aufgeworfenen Fragen. Es mag große Verbesserungspotentiale im Fach geben. Dennoch denke ich, dass die herkömmliche Statistik so viele Erfolge aufzuweisen hat, dass schon vermehrtes gemeinsames und offensives Auftreten in einem höheren Stellenwert resultieren wird. Diese Einschätzung basiert auf der mehrjährigen Beobachtung der Strategien der mehr oder weniger erfolgreichen Akteure im innerwissenschaftlichen Wettbewerb.

Zweitens möchte ich mich bei den Kollegen Rendtel und Gertheiss für klärende Gespräche, Informationen und konkrete Vorschläge herzlich bedanken

## Ist Statistik unter einem Dach erfolgreich?

*Christine Müller*

Dieser Beitrag liefert eine Einschätzung zu den Gründungsumständen und den Erfolgen der Deutschen Arbeitsgemeinschaft Statistik, kurz DAGStat. Diese Einschätzung wird gespeist durch persönliche Erfahrungen als Vorsitzende der DAGStat von 2013 bis 2019, was die Sicht einer Statistikerin und nicht einer Wissenschaftshistorikerin ist.

### Ziele der DAGStat

Die DAGStat wurde am 17. Juni 2005 mit folgenden Zielen gegründet (siehe auch Deutsche Arbeitsgemeinschaft Statistik 2022a):

- Vernetzung der im Bereich der Statistik tätigen Wissenschaftler und Anwender,
- Verbesserung der Wahrnehmung der Statistik in der Öffentlichkeit,
- Verbesserung des Standes der Statistik in den Wissenschaften.

Was war aber die Ausgangssituation für diese Gründung?

### Die Situation der Statistik zur Zeit der Gründung der DAGStat

In den siebziger Jahren waren im Zuge des Ausbaus der Universitäten in Westdeutschland und Westberlin neben den Lehrstühlen in Wirtschaftsstatistik und Biometrie auch viele neue Lehrstühle in Mathematischer Statistik an Mathematikfachbereichen entstanden. Zusätzlich wurde in dieser Zeit der Statistikfachbereich in Dortmund gegründet. Die Schaffung speziell der Statistik-Stellen wurde insbesondere durch den Contergan-Skandal begünstigt, bei dem mangelnde Statistikexpertise dazu geführt hatte, dass die Ursache der gehäuft auftretenden Missbildungen erst sehr spät gefunden wurde. Damit waren die Siebziger Jahre eine richtige Blütezeit der Statistik.

Das endete mit der Sparpolitik in den neunziger Jahren nach der Wende. Weil Statistiker eher am Rande ihrer Fachbereiche standen, wurde oft der Sparstift bei der Statistik angesetzt. Zugleich boomte die Finanzmathematik, die weitere Stellen der Statistik okkupierte. So wurde die Statistik an der FU Berlin von vorher ca. 6 Professuren in den Wirtschaftswissenschaften und der Mathematik auf wenig mehr als zwei Stellen reduziert.

Vom Boom der Bioinformatik um das Jahr 2000 konnte die Statistik zunächst auch nicht viel profitieren und verlor noch weitere Stellen. Somit musste die Statistik seit den neunziger Jahren stark um ihre Existenz an den akademischen Einrichtungen kämpfen.

Im Gegensatz zu den angelsächsischen Ländern, die viele Statistik oder Maths- & Stats-Fachbereiche besitzen, ist die Statistik in Deutschland über verschiedene Fachbereiche zersplittert und damit ohne mächtigen Fürsprecher. Neben der Zersplitterung der Statistik an den Universitäten ist sie auch noch auf verschiedenste Gesellschaften verteilt. Es gibt nicht wie in Großbritannien die Royal Statistical Society oder in den USA die American Statistical Association, die die verschiedenen Statistik-Disziplinen vereint und damit besser vertreten kann.

**Figure 1:**
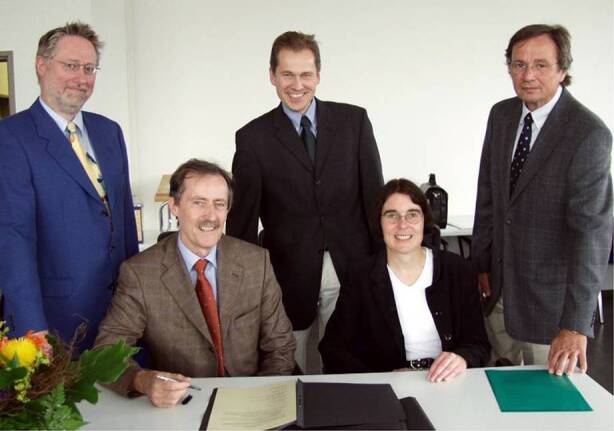

Dadurch entstand die Idee der Gründung einer Dachgesellschaft Statistik, die vor allem von Göran Kauermann, Karl Mosler von der Deutschen Statistischen Gesellschaft (DStatG) und Joachim Röhmel von der Deutschen Region der Internationalen Biometrischen Gesellschaft (IBS-DR) vorangetrieben wurde. Mitglieder dieser Dachgesellschaft sollten nur Gesellschaften und Institutionen mit Statistik-Bezug sein. Bei der Gründung waren dann neben der DStatG und der IBS-DR noch die Gesellschaft für Klassifikation vertreten durch Claus Weihs und die DMV-Fachgruppe Stochastik vertreten durch Christine Müller beteiligt, siehe Abb. 1.

Göran Kauermann wurde der erste Vorsitzende und hat maßgeblich die DAGStat geprägt. Mittlerweile hat die DAGStat 14 Mitglieder, darunter auch das Statistische Bundesamt. Das Wachsen der DAGStat auf 14 Mitgliedsgesellschaften ist schon ein großer Erfolg. Was ist aber mit den unter 1. genannten Zielen der DAGStat?

### Vernetzung der im Bereich der Statistik tätigen Wissenschaftler und Anwender

**Figure 2:**
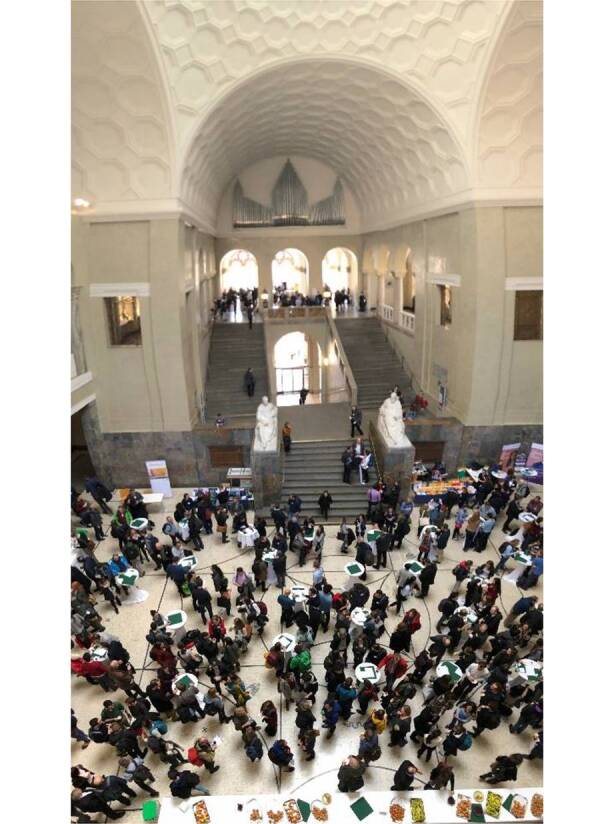

Göran Kauermann etablierte die erfolgreiche DAGStat-Tagung als wichtige gemeinsame Tagung aller in der Statistik tätigen Wissenschaftler und Anwender von Statistik. Nach dem Start 2007 an der damaligen Universität von Göran Kauermann in Bielefeld finden diese Tagungen seitdem alle drei Jahre statt, nämlich 2010 in Dortmund, 2013 in Freiburg, 2016 in Göttingen und 2019 in München (siehe Deutsche Arbeitsgemeinschaft Statistik 2022b; und Abb. 2). Diese Tagung ist mittlerweile sehr beliebt und hatte in München fast 900 Teilnehmer. Einige der Mitgliedsgesellschaften führen im Rahmen der DAGStat-Konferenz ihre Jahrestagungen durch.

**Figure 3:**
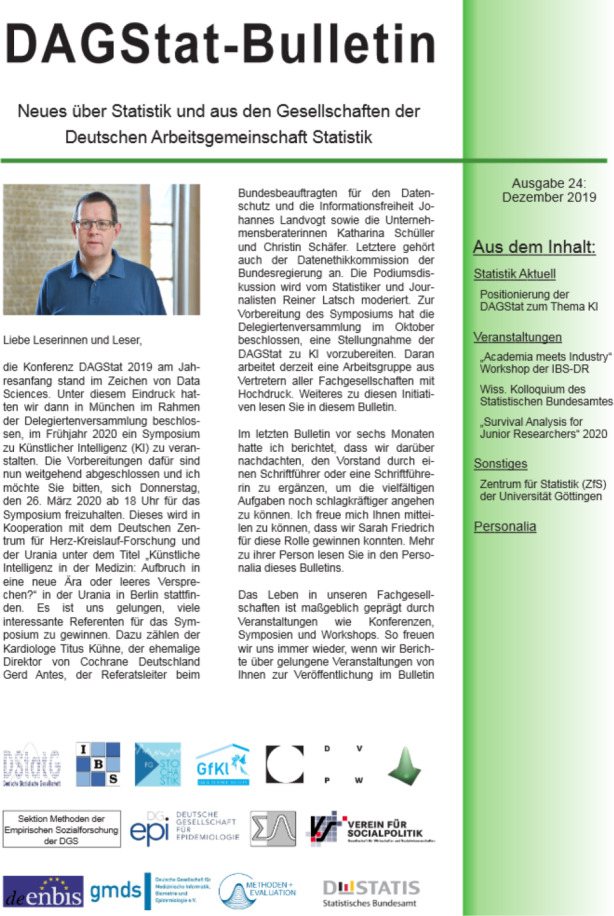

Göran Kauermann führte auch das DAGStat-Bulletin (siehe Deutsche Arbeitsgemeinschaft Statistik 2022c; und Abb. 3) ein, das halbjährlich über Aktivitäten der DAGStat berichtet und Arbeitsfelder von Statistikern vorstellt. Dieses Bulletin richtet sich somit auch an eine breitere Öffentlichkeit, da Öffentlichkeitsarbeit ein weiteres Ziel der DAGStat ist.

### Verbesserung der Wahrnehmung der Statistik in der Öffentlichkeit

**Figure 4:**
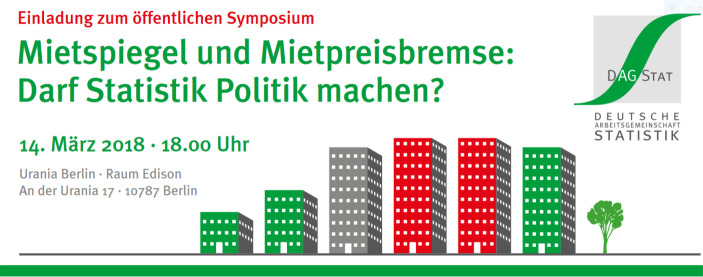

Um eine breitere Öffentlichkeit zu erreichen, wurden seit 2008 insgesamt 10 Symposien durchgeführt. Um auch Bundespolitiker zu erreichen, fanden sie an verschiedenen Institutionen in Berlin statt. Sie hatten die folgenden Themen (siehe auch Deutsche Arbeitsgemeinschaft Statistik 2022d):

- 2008 – Die Zukunft des Pflegebedarfs in Deutschland – Demographischer Wandel, medizinischer Fortschritt & ökonomische Vorsorge
- 2009 – Hartz IV – Die Folgen von Hartz IV
- 2010 – Die Fettleibigkeit der Deutschen – Empirisch-statistische Aspekte
- 2011 – Möglichkeiten und Grenzen des Zensus 2011 – Gesellschaft mit beschränkter Information?
- 2012 – Migranten in Deutschland – Zahlen – Fakten – Zusammenhänge
- 2013 – Gesundheitsrisiken – Was bedroht unser Leben wirklich?
- 2014 – Wie sehr regieren uns Indikatoren – Staatsschulden, Wohlstand und Statistik
- 2015 – Big Data – Big Brother oder Big Chances?
- 2017 – Ist Bildung messbar?
- 2018 – Mietspiegel und Mietpreisbremse: Darf Statistik Politik machen? Siehe Abb. 4.

Diese Symposien haben unterschiedlich gut die allgemeine Bevölkerung erreicht.

So erschien über das Symposium „Ist Bildung messbar“ ein Artikel in der Berliner Tageszeitung „Der Tagesspiegel“. Aber dieses Symposium hatte nur wenige Teilnehmer. Das war beim Symposium „Mietspiegel und Mietpreisbremse: Darf Statistik Politik machen?“ ganz anders, bei dem zahlreiche Mitglieder von Mietervereinen und Vermietervereinen teilnahmen. Außerdem wurde es von Urania-Mitgliedern besucht, da es erstmalig in Kooperation mit der Berliner Urania durchgeführt wurde. Andere Symposien erreichten allerdings nicht so gut die breite Öffentlichkeit und dienten mehr dem Austausch von Spezialisten. Eventuell wird das in Zukunft besser, wenn die Symposien in Kooperation mit der Urania stattfinden.

### Verbesserung des Standes der Statistik in den Wissenschaften

Bei dem weiteren Ziel der DAGStat, den Stand der Statistik in den Wissenschaften zu verbessern, stellt sich das Problem, dass die Statistik als eigenständige wissenschaftliche Disziplin gar nicht richtig erfasst wird.

Was nicht erfasst wird, existiert nicht.

Es ist fast ein Treppenwitz, dass selbst beim Statistischen Bundesamt (DESTATIS) die Statistik als akademisches Fachgebiet bis vor kurzem nur sehr rudimentär erfasst wurde, siehe Tab. 1. Diese Tabelle gibt nur die Erfassung bezüglich des Personals an Hochschulen wieder. Bei der Klassifikation von Studierenden gibt es überhaupt keine Rubrik mit Statistik. Damit werden die verschiedenen Statistik-Studiengänge in Deutschland einfach gar nicht erfasst.

**Table 1:** 

Lehr- und Forschungsbereich	
Rechts‑, Wirtschafts- und Sozialwissenschaften	Ökonometrie
Psychologie	–
Mathematik, Naturwissenschaften	Mathematische Statistik/Wahrscheinlichkeitsrechnung
Humanmedizin/Gesundheitswissenschaften	Epidemiologie, Medizinische Statistik und Dokumentation
Ingenieurwissenschaften	–

Somit ist es auch nicht verwunderlich, dass die Statistik auch bei den Fachkollegien der Deutschen Forschungsgemeinschaft (DFG) kaum vertreten ist, siehe Tab. 2. Dadurch hat es die Statistik-Forschung in Deutschland sehr schwer, da die Fachkollegien entscheiden, welche Forschungsprojekte gefördert werden. Insbesondere jede Statistik-Forschung, die weder Ökonometrie, Biometrie noch Mathematische Statistik ist, hat recht geringe Chancen über die DFG gefördert zu werden.

**Table 2:** 

Fachkollegium	Fach	Anzahl der Kollegiaten
Psychologie	Keine Statistik	0 von 12
Wirtschaftswissenschaften	Statistik und Ökonometrie	2 von 14
Medizin	Epidemiologie, Medizinische Biometrie, Medizinische Informatik	3 von 85
Mathematik	Keine Statistik	0 von 8
Informatik	Keine Statistik	0 von 21

Die DAGStat hat immer wieder auf diesen Missstand hingewiesen. Nachdem sie 2015 das Vorschlagsrecht für die Benennung von Kandidierenden für die DFG-Fächer „Statistik und Ökonometrie“ und „Epidemiologie, Medizinische Biometrie, Medizinische Informatik“ erhielt, wird sie allgemein ernster genommen. So wurde sie in letzter Zeit bei den Konsultationen zur Revision der Fächerklassifikation beim Statistischen Bundesamt und bei der DFG miteinbezogen.

Nach einem ersten vergeblichen Versuch, zumindest die Fächerklassifikation beim Statistischen Bundesamt zu ändern, gibt es jetzt einen Erfolg: Sowohl in der Personalstatistik als auch in der Studierenden-Statistik wird es in Zukunft in der Fächergruppe „Mathematik, Naturwissenschaften“ die Rubrik „Statistik“ geben.

**Figure 5:**
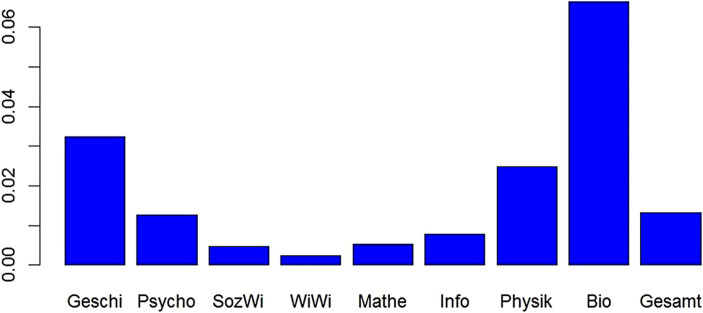

Bezüglich der Fächerklassifikation bei der DFG gab es am 22.11.2017 ein Rundgespräch mit Vertretern der DFG und der DAGStat, bei dem die Grafik in Abb. 5 vorgestellt wurde. Diese Grafik zeigt, dass bestimmte Fächer bei den Kollegiaten, die über die Forschungsanträge entscheiden, überrepräsentiert und andere unterrepräsentiert sind. So sind die Mathematik und die Wirtschaftswissenschaften unterrepräsentiert. Das hat sicherlich historische Gründe, da die überrepräsentierten Fächer solche Fächer sind, die schon immer auf zusätzlich Mittel für ihre Forschung angewiesen waren, wie Archäologie, Medizin oder die Ingenieurwissenschaften. Da aber heutzutage ein Großteil des wissenschaftlichen Personals über die DFG finanziert wird und Professorengehälter sich nach eingeworbenen Drittmitteln richten, besteht die Gefahr, dass die historisch gewachsene Zuordnung von Kollegiaten die Fächer benachteiligt, die früher ohne Drittmittel gut Forschung betreiben konnten. Auch wenn die Überrepräsentanz und Unterrepräsentanz von Fächern von dem Mathematik-Vertreter der DFG als nicht relevant abgetan wurde, gab es danach eine Änderung bei den Anzahlen der Kollegiate: Die Anzahl der Kollegiate in der Mathematik wurde von 8 auf 10 erhöht und im Fach „Epidemiologie, Medizinische Biometrie, Medizinische Informatik“ wurde die Anzahl von 3 auf 4 verbessert, wobei dort zusätzlich noch die Medizinische Informatik herausgenommen wurde. Damit bleibt aber weiterhin die Situation der Statistik unbefriedigend. Aber vielleicht ändert sich noch etwas, nachdem die Fächerklassifikation beim Statistischen Bundesamt geändert wurde.

Ein Resultat des Rundgesprächs war ein Rundgespräch zu einem Schwerpunkt-Programm Datenwissenschaften bei der DFG. Bei der DFG fand auch ein Rundgespräch zu einem Schwerpunkt-Programm im Bereich Maschinenbau und Datenwissenschaften statt, wo der Satz fiel: „Ich befürchte, wir können Statistik nicht vermeiden.“ Dieser Satz zeigt deutlich, mit welchem Zwiespalt Wissenschaftler anderer Disziplinen und insbesondere der Ingenieurwissenschaften der Statistik gegenüberstehen. Solche Wissenschaftler sehen schon, dass Statistik im Zeitalter von Big Data und Data Science immer wichtiger wird. Sie ziehen aber vorhandene Kooperationen mit der Informatik vor. Damit besteht die Gefahr, dass das Gebiet der Datenwissenschaften insbesondere in den Ingenieurwissenschaften vollständig von der Informatik ohne richtigen statistischen Sachverstand dominiert wird.

**Figure 6:**
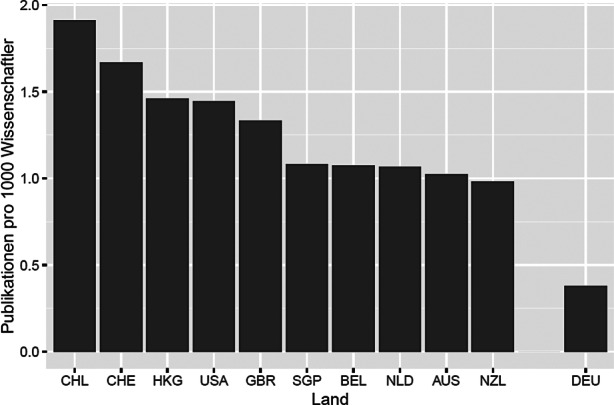

Das ist aber ein deutsches Phänomen. Wie weit Deutschland bereits im Bereich Statistik abgehängt ist, wurde in Szugat et al. (2017) gezeigt. Bei der Anzahl von Publikationen in hochrangigen internationalen Statistik-Zeitschriften pro 1000 Wissenschaftler liegt Deutschland innerhalb von 27 betrachteten Ländern abgehängt auf dem 19. Platz nach Ländern wie Chile, Schweiz, Singapur und Niederlanden, siehe Abb. 6.

### Fazit

Bezüglich der Vernetzung von im Bereich Statistik tätigen Personen ist die DAGStat erfolgreich. Die Öffentlichkeitsarbeit kann noch verbessert werden und ist mit den Symposien auf einem guten Weg. Der Stand in den Wissenschaften ist durch die Modifikation der Fächerklassifikation beim Statistischen Bundesamt verbessert worden. Aber das kann nur ein erster Anfang sein. Insbesondere bei der DFG müssten noch Änderungen passieren, damit Deutschland bezüglich Statistik nicht ganz abgehängt wird. Dabei wird im Zeitalter von Big Data und Data Science die Statistik immer wichtiger, siehe Kauermann und Seidl (2018), Kauermann (2020). Es könnte aber passieren, dass statistische Aspekte gegenüber informatischen Aspekten zu sehr vernachlässigt werden. Somit ergeben sich viele wichtige und nicht ganz einfache Aufgaben für den neuen DAGStat-Vorsitzenden Tim Friede. Bezüglich der aktuellen Aktivitäten der DAGStat siehe das DAGStat-Bulletin vom Dezember 2019.

### Danksagung

Ich danke Göran Kauermann und Tim Friede für das kritische Durchlesen dieses Textes und für deren sehr hilfreiche Anregungen.

## Amtliche Statistik – Informationelle Infrastruktur in der digitalen Transformation

*Jürgen Chlumsky, Markus Zwick*

### Einleitung

Amtliche Statistik hat eine sehr lange Tradition. Diente sie zunächst ausschließlich ganz bestimmten Zwecken, wie etwa der Aufstellung eines Heeres oder der Steuererhebung, so gibt sie heute in allen entwickelten Gesellschaften nicht nur den Regierungen und der Verwaltung einen Überblick über den jeweiligen Stand der ökonomischen und sozialen Entwicklungen. Vielmehr hat die amtliche Statistik darüber hinaus einen allgemeinen Informationsauftrag. Ihre Informationen stehen der gesamten Öffentlichkeit unentgeltlich zur Verfügung und dienen der Willensbildung der demokratischen Gesellschaft.

Zur langen Tradition der amtlichen Statistik gehören gleichermaßen Kontinuität und Wandel. Nur eine gewisse Kontinuität des Programms und der Methodik ermöglicht Vergleiche im Zeitablauf, nur eine angemessene Reaktion auf sich permanent wandelnde Informationsbedarfe der Gesellschaft, der Wissenschaft und der Politik rechtfertigt eine öffentliche Finanzierung.^14^

Hauptproduzenten amtlicher Statistiken sind in der Bundesrepublik Deutschland die Statistischen Ämter des Bundes und der Länder. Darüber hinaus erstellen aber etwa auch die Deutsche Bundesbank für den Geld- und Kreditbereich und die Bundesagentur für Arbeit über den Arbeitsmarkt amtliche Statistiken. Neben diesen Institutionen sind auch die Ministerien, insbesondere mit ihren nachgeordneten Behörden, als amtliche Datenproduzenten tätig. Zu nennen sind hier als Beispiel das Robert-Koch Institut als nachgeordnete Behörde des Bundesministeriums für Gesundheit oder das Kraftfahrtbundesamt als nachgeordnete Behörde des Bundesministeriums für Verkehr und digitale Infrastruktur. Kennzeichnend ist auch hier, dass die entsprechenden Daten bzw. Statistiken in der Regel gesetzlich angeordnet und öffentlich finanziert sind.

Auf drei Entwicklungen der letzten Jahrzehnte, die ganz erhebliche Auswirkungen auf das Programm, die Methodik und die Rolle der amtlichen Statistik in Deutschland hatten, wird hier im Folgenden näher eingegangen.

Zum einen war dies das Urteil des Bundesverfassungsgerichts in Folge des Widerstands gegen die geplante Volkszählung 1983, in dem das Recht auf informationelle Selbstbestimmung als Grundrecht anerkannt worden ist.^15^ Dieses Urteil ist noch heute prägend für das Handeln der amtlichen Statistik und hat auch wesentlich die Entwicklung der Europäischen Datenschutzgrundverordnung (DSGVO) mit beeinflusst.

Des Weiteren hat die Europäisierung der amtlichen Statistik diese in Deutschland verändert. Das fachliche Programm der deutschen amtlichen Statistik ist inzwischen überwiegend europäisch bestimmt, die Integration der Statistiksysteme innerhalb des Europäischen Statistischen Systems (ESS) weit fortgeschritten. Eurostat, das Statistische Amt der Europäischen Union, hat die Aufgabe, die nationalen Statistiken, die als europäische Gemeinschaftsstatistiken entstehen, zu harmonisieren und damit vergleichbar auszugestalten. Eurostat ist dabei in der Regel nicht selbst Datenproduzent, sondern Koordinator der Statistikproduktion in den Mitgliedstaaten und nimmt insoweit damit eine ähnliche Rolle ein wie das Zentralbureau schon für den deutschen Zollverein, das Kaiserliche Statistische Amt im Jahre 1872 für das damals neugegründete Deutsche Reich und das Statistische Bundesamt für die föderal organisierte Bundesstatistik.

Eine dritte große Veränderung der amtlichen Statistik geht mit der Nutzung und Verknüpfung von bereits für andere Zwecke verfügbaren Daten einher. Stichworte sind hier Registerdaten, Big Data, aber auch Data Science und Artificial Intelligence.

### Informationelle Selbstbestimmung und Datenzugang für die Wissenschaft

Das Urteil des Bundesverfassungsgerichts zur informationellen Selbstbestimmung von 1983 prägt die deutsche amtliche Statistikproduktion bis heute in außerordentlich starkem Maße. Das Bundesverfassungsgericht wurde 1983 im Zuge der politischen Auseinandersetzung um die Volkszählung 1983 im Rahmen verschiedener Einwendungen angerufen. Im sogenannten Volkszählungsurteil kam das Bundesverfassungsgericht zu dem Schluss, einzelne Vorschriften des Volkszählungsgesetzes 1983 berücksichtigten die notwendige Zweckgebundenheit der Eingriffe in das Persönlichkeitsgrundrecht nach § 2 Abs. 1 GG nicht in sachgerechter Weise. Insbesondere das damals vorgesehene Rückspielen von nicht-anonymisierten Einzeldaten aus dem Zensus an die Einwohnermeldeämter wurde als verfassungsrechtlich problematisch angesehen. Das Bundesverfassungsgericht unterstrich in seinem Urteil aber auch sehr eindeutig die Befugnis des Staates, für seine Informationsbedarfe auch Freiheitsrechte auf gesetzlicher Basis einzuschränken.^16^

Zu verstehen ist die seinerzeit sehr hitzig geführte öffentliche Diskussion um die Volkszählungen 1983 und 1987 in ihrer Schärfe heute nur im Kontext anderer damals heftig geführter politischer Diskussionen. Der Doppelbeschluss zur atomaren Rüstung von 1979 führte zum Verschmelzen der Friedensbewegung und der Bewegung gegen Atomkraftwerke. Das generelle Misstrauen gegen den Staat richtete sich dann auch gegen die Volkszählung, Tendenzen hin zu einem Überwachungsstaat – wie in George Orwells 1948 erschienenem Bestseller 1984 beschrieben^17^ – wurden befürchtet^18^. Diese damalige abstrakte Besorgnis ist heute mit den Realitäten von Google, Facebook und Amazon zum Teil schwer nachzuvollziehen.

In starkem Maße von der Volkszählungsdiskussion überlagert war in diesen Jahren die Frage, wie öffentlich finanzierte Daten der empirisch arbeitenden Wissenschaft besser verfügbar gemacht werden könnten. Eine erste Antwort hierauf wurde bereits mit dem überarbeiteten Bundesstatistikgesetz 1981 formuliert. Dieses stellte sich aber als wenig zielführend heraus. Es zeigte sich schnell, dass die hier vorgesehene Form der Weitergabe von ursprünglich personenbezogenen Daten in vollständig anonymisierter Form für wissenschaftliche Anwendungen zu restriktiv war. Bereits im Jahre 1987 wurde das Bundesstatistikgesetz (BStatG) daher in seinem § 16, der die statistische Geheimhaltung regelt, erweitert. § 16 Abs. 6 BStatG erlaubt es seither der Wissenschaft Einzeldaten zur Verfügung zu stellen, wenn diese nur mit einem unverhältnismäßig großen Aufwand an Zeit, Kosten und Arbeitskraft zugeordnet werden können. Zu Beginn der neunziger Jahre entstanden auf dieser rechtlichen Grundlage die ersten faktisch anonymisierten Datensätze des Mikrozensus und der Einkommens- und Verbrauchsstichprobe.^19^

Dies war der Startpunkt einer dynamischen Entwicklung. Als ein wesentlicher Treiber dieser Entwicklungen ist hier das im Sommer 1999 in Wiesbaden vom Statistischen Bundesamt veranstaltete Symposium zum Thema „Kooperation zwischen Wissenschaft und amtlicher Statistik – Praxis und Perspektiven“ zu sehen. Die Teilnehmenden des Symposiums schlugen in einem Thesenpapier vor, eine Kommission einzurichten, die Lösungsvorschläge zu den inhaltlichen, organisatorischen und ressourcenbezogenen Fragen der Zusammenarbeit zwischen Wissenschaft und amtlicher Statistik erarbeitet.^20^ Der Weitsicht des Bundesministeriums für Forschung und Entwicklung und insbesondere der damaligen Ministerin Edelgard Bulmahn ist die dann zügig erfolgte Einrichtung der Kommission zur Verbesserung der informationellen Infrastruktur zwischen Wissenschaft und Statistik (KVI) zu verdanken. Das aus dem Jahr 2001 stammende KVI-Gutachten empfahl u. a. die Etablierung eines Rats für Sozial- und Wirtschaftsdaten (RatSWD) sowie die Einrichtung von Forschungsdatenzentren bei den öffentlichen Datenproduzenten.^21^ Die Datenlandschaft in Deutschland veränderte sich in der Folge nachhaltig. Zu verdanken ist dies wesentlich den beiden Leitern der Kommission, Professor Dr. Hans Jürgen Krupp und Johann Hahlen, Staatssekretär a. D. und seinerzeit Präsident des Statistischen Bundeamtes.^22^

Mittlerweile, nach einer rund dreißigjährigen Diskussion zwischen den öffentlichen Datenproduzenten und der empirischen Wissenschaft, ist ein Zugang zu Einzeldaten der für die empirische Forschung wesentlichen amtlichen Statistiken Standard. Die Diskussion über den Zugang zu mit öffentlichen Mitteln produzierten Daten wurde zunächst in einem Gründungsausschuss und im weiteren Verlauf im Rat für Sozial- und Wirtschaftsdaten (RatSWD) geführt. Finanziert durch das Bundesministerium für Bildung und Forschung (BMBF) hat der RatSWD (www.ratswd.de)^23^, paritätisch besetzt mit empirisch arbeitenden Wissenschaftler/innen und Datenproduzenten, die sogenannte informationelle Infrastruktur deutlich ausgebaut. Der Forschung steht heute eine hochentwickelte und nachhaltige Forschungsinfrastruktur zur Verfügung. Ein dezentrales Netzwerk von 38 vom RatSWD akkreditierten Forschungsdatenzentren (FDZ) ermöglicht einen in der Regel kostengünstigen und einfachen Zugang zu einer Vielzahl von Forschungsdaten (https://www.konsortswd.de/datenzentren/alle-datenzentren/)^10^. Zu den über die FDZ erhältlichen Forschungsdaten gehören dabei auch umfangreiche Metadateninformationen, sodass der Generierungsprozess der Daten transparent nachvollzogen werden kann. Mit der Etablierung des Konsortiums für die Sozial‑, Verhaltens‑, Bildungs- und Wirtschaftswissenschaften (www.KonsortSWD.de) mit Beschluss der Wissenschaftskonferenz von Bund und Ländern vom Juni 2020 wird der RatSWD künftig über das KonsortSWD im Rahmen der Nationalen Forschungsdateninfrastruktur (NFDI) gefördert.

Trotz aller Fortschritte gelingt es aber nach wie vor nicht, die Datenbedarfe, die sich aus der empirischen Wirtschafts- und Sozialforschung ergeben, vollständig zu befriedigen. Die Aufgabe erinnert an den Wettlauf von Hase und Igel. Neue Forschungsansätze und neue Forschergenerationen fordern neue Verbreitungsinhalte und -wege. Datensätze, die von der jeweils etablierten empirischen Wissenschaft genutzt wurden, sind in der jeweiligen Form für die nächste Wissenschaftsgeneration zum Teil nur noch bedingt von Interesse, das Informationspotential vielfach auch weitgehend ausgeschöpft.

Die Bereitstellung faktisch anonymisierter Einzeldaten begann in den neunziger Jahren des letzten Jahrhunderts zunächst mit den Querschnittserhebungen des Mikrozensus und der Einkommens- und Verbraucherstichprobe. Später wurden wirtschaftsstatistische Daten einbezogen, dann auch komplexere Datensätze wie Paneldaten. Eine Neuerung und für die amtliche Statistik zunächst durchaus eine Herausforderung war es, Einzeldaten verschiedener Erhebungen quer und über die Zeit zusammenzuspielen, wie etwa die AFiD Panel Daten.^24^ Heute geht es darum, Befragungsdaten mit Registerdaten und neuen digitalen Daten zu verknüpfen. Von Seiten der Wissenschaft wird die Forderung gestellt, amtliche Befragungsdaten, zum Beispiel aus dem Mikrozensus, etwa mit Sensordaten der Mobilfunkgeräte oder Fitnessarmbänder zu kombinieren und verfügbar zu machen.

Die Diskussionen über den Zugang zu personenbezogenen Daten der amtlichen Statistik ist weiterhin in Bewegung. Zum einen ist der von der amtlichen Statistik unternommene Versuch des Ausgleichs zwischen den Verfassungszielen Wissenschaftsfreiheit und informationeller Selbstbestimmung umstritten, zum anderen haben Gesetzesänderungen neue Grundlagen geschaffen. Hier ist die Erweiterung des § 16 Abs. 6 BStatG zu nennen, eine Regelung die nun den Zugang zu formal anonymisierten Daten erlaubt. Aber auch mit der Europäischen Datenschutzgrundverordnung sind neue Aspekte hinzugekommen.^25^

Als amtlicher Datenproduzent muss man es aushalten können, dass die Nutzergruppe Wissenschaft nie zufrieden ist. Sie kann es gar nicht sein, bleiben doch mit jeder Runde Dateninnnovation immer noch viele Fragen offen. Am aktuellen Rand ist der Datenproduzent ständig der Entwicklung hinterher, eine Betrachtung über die Zeit hingegen zeigt, dass dennoch viel erreicht wurde.

### Das Europäische Statistische System (ESS)

Vielen Datennutzern ist gar nicht bewusst, dass die deutsche amtliche Statistik mittlerweile im Grunde europäische Statistik ist. Der Großteil der in Deutschland durch den Verbund der Statistischen Ämter des Bundes und der Länder durchgeführten Statistiken sind sogenannte Gemeinschaftsstatistiken und diese beruhen auf einer europäischen Rechtsgrundlage.

Die Verordnung (EG) Nr. 223/2009 des europäischen Parlaments und des Rates über die Gemeinschaftsstatistiken bildet die Rechtsgrundlage für die Erstellung des Europäischen Statistischen Programms und ist damit die Grundlage und Rahmenverordnung im Europäischen Statistischen System (ESS).^26^ Ergänzt wird diese Verordnung durch spezifische rechtliche Regelungen für die jeweiligen Gemeinschaftsstatistiken.

Das ESS ist eine Partnerschaft zwischen den statistischen Institutionen der Gemeinschaft, also Eurostat in Luxemburg, einer Generaldirektion der Europäischen Kommission, den nationalen statistischen Ämtern (NSÄ) und anderen einzelstaatlichen Stellen, die in den einzelnen Mitgliedstaaten für die Entwicklung, Erstellung und Verbreitung europäischer Statistiken zuständig sind, wie in Deutschland etwa die Bundesagentur für Arbeit. Europäisch geregelt ist insbesondere der Rahmen zur Produktion qualitativ hochwertiger und europäisch vergleichbarer Statistiken. Wichtige Elemente sind hierbei die fachliche Unabhängigkeit, die Unparteilichkeit, die Objektivität sowie die Zuverlässigkeit der NSÄ im ESS. Eckpfeiler des gemeinsamen Qualitätsrahmens bildet ein von den Mitgliedstaaten verabschiedeter Verhaltenskodex für europäische Statistiken. Dieser Code of Practice (CoP) ist ein Instrument der Selbstregulierung und beruht auf sechzehn Grundsätzen für das institutionelle Umfeld, die statistischen Prozesse und den statistischen Output.^27^

Das ESS strebt auf der Grundlage gemeinsamer Qualitätsmaßstäbe insbesondere eine Vergleichbarkeit der europäischen Statistiken an. Dies ist eine anspruchsvolle Aufgabe, nicht nur für Eurostat, sondern vor allem für die Mitgliedstaaten, da viele Kompromisse notwendig sind um tatsächlich vergleichbare Daten zu produzieren. Für die amtliche Statistik in Deutschland bedeutete dies in der Vergangenheit und bedeutet auch für die Zukunft eine Weiterentwicklung der Konzepte und Methoden insbesondere in den Fällen, in denen diese der deutschen Statistiktradition nicht entsprechen.^28^

In der deutschen amtlichen Statistik wird bei der Konzeption einer amtlichen Statistik vielfach auf Daten zurückgegriffen, die in der Verwaltung bereits vorliegen. Ein Beispiel ist das Konzept von Erwerbs- und Arbeitslosigkeit, das traditionell in Deutschland von der Definition der Bundesagentur für Arbeit (BA) geprägt ist. Die Zahl der Arbeitslosen entspricht der bei der BA arbeitslos Gemeldeten, also der registrierten Arbeitslosen. Wer sich also aus welchen Gründen auch immer nicht arbeitslos meldet, geht nicht in die Arbeitslosenstatistik ein. Im Zuge der europäischen Harmonisierung einigte man sich dagegen auf das ILO-Konzept. Gemäß dem Labor-Force-Konzept der International Labour Organization (ILO) sind alle Personen arbeitslos bzw. erwerbslos, die ohne Arbeitsverhältnis sind, dem Arbeitsmarkt zur Verfügung stehen und sich um einen Arbeitsplatz bemühen.^29^ Die Erhebung erfolgt im Rahmen einer gemeinschaftlichen Arbeitskräfteerhebung, die in Deutschland als Unterstichprobe in den Mikrozensus integriert ist.^30^

Gegenwärtig wird der Unternehmensbegriff auch in Deutschland an den europäisch harmonisierten Unternehmensbegriff angepasst. Definiert war ein Unternehmen in der deutschen amtlichen Statistik bislang als kleinste rechtliche Einheit, also eine juristische oder natürliche Person, die eine wirtschaftliche Tätigkeit selbstständig ausübt. Ein Unternehmen gemäß der EU-Einheitenverordnung ist hingegen nun die „kleinste Kombination rechtlicher Einheiten, die eine organisatorische Einheit zur Erzeugung von Waren und Dienstleistungen bildet und […] über eine gewisse Entscheidungsfreiheit verfügt.“ Somit kann ein Unternehmen auch aus mehreren rechtlichen Einheiten bestehen. Das Unternehmen als kleinste rechtliche Einheit war administrativ klar abgegrenzt, der EU-Unternehmensbegriff ist es nicht. Dafür ist der Unternehmensbegriff nun im ESS vergleichbar, unabhängig von nationalen Rechtsformen.

Auch der in der deutschen amtlichen Statistik bisher verwendete Bevölkerungsbegriff wird absehbar aufgrund europäischer Harmonisierung angepasst werden. Die deutsche Bevölkerung definiert sich administrativ nach dem § 12 Abs. 2 Melderechtsrahmengesetz. Wer gemeldet ist, der gehört zur deutschen Bevölkerung. Der europäische Bevölkerungsbegriff basiert dagegen auf dem üblichen Aufenthaltsort einer Person.^31^

Die Definitionen von Arbeits- bzw. Erwerbslosigkeit, Unternehmen wie auch Bevölkerung folgten beziehungsweise folgen in Deutschland einer sehr praktikablen Abgrenzung. Sie basieren auf einer rechtlichen Grundlage, die nicht primär Zwecken der Statistik dient, die statistischen Einheiten sind vielfach in Registern erfasst und können sekundärstatistisch genutzt werden. Für Gemeinschaftsstatistiken sind diese Abgrenzungen aber häufig nicht vergleichbar. Die europäischen Definitionen hingegen sind dies und sie beschreiben die Realität auch im Sinne des Adäquationsprinzips^32^ oft besser.

Wie diese wenigen Beispiele zeigen, hat das ESS einen erheblichen Einfluss, wie wir ‚Realität‘ in Deutschland statistisch abbilden und so auch dazu beitragen, im öffentlichen Bewusstsein Realität zu schaffen. Das ESS ist damit und darüber hinaus innovationsgebend. Auch die aktuellen Entwicklungen im Hinblick auf die Nutzung neuer digitaler Daten und Methoden (Big Data) sind insbesondere durch Aktivitäten im ESS getrieben, aber dazu im nächsten Kapitel mehr.

### Neue digitale Daten und Methoden

Mit den neuen Daten der digitalen Revolution und den damit möglichen erweiterten statistischen Methoden, z. B. beim maschinellen Lernen, haben sich wesentliche Grundlagen der qualitativ hochwertigen Datenproduktion verändert.

Insbesondere auch die Frage der Adäquation, also die sachgerechte numerische Abbildung ‚realer‘ Gegebenheiten, stellt sich im digitalen Datenzeitalter anders. Bei der traditionellen Herangehensweise, die vor allem durch die Frankfurter Schule geprägt wurde,^33^ wird ein theoretisches Konstrukt durch Operationalisierung messbar gestaltet und auf dieser Grundlage werden die interessierenden Daten produziert. Dies funktioniert im digitalen Zeitalter so nicht mehr. Eine Veränderung bahnte sich schon mit der intensiveren Nutzung administrativer Daten in der amtlichen Statistik und im Weiteren in den Analysen der empirischen Sozialwissenschaften an. Bei allen Daten, die sekundärstatistisch genutzt werden, steht das Adäquationsproblem nicht am Anfang der Datenproduktion, sondern am Ende. Administrative wie neue digitale Daten liegen vor, generiert im Rahmen eines nicht-statistischen Prozesses. Die Kunst des Statistikers ist es nun zu verstehen, welcher Teil dieser Daten eine interessierende Realität empirisch adäquat abbildet und welcher Teil im Untersuchungskontext unbrauchbar ist.

Bei neuen digitalen Daten ist dies eine anspruchsvolle und schwierige Aufgabe. Zum einen ist der Zugang zu neuen digitalen Daten, die meist privat produziert werden, komplex, manchmal kaum möglich und häufig sehr teuer.^34^ Zum anderen sind die Daten, wie z. B. Mobilfunkdaten,^35^ zwar über den Informationsmarkt zugänglich, dies aber in einer vorproduzierten Form und häufig mit sehr beschränktem Zugang zu den erläuternden Informationen des datengenerierenden Prozesses, also den Metadaten. Gleichwohl existieren durchaus Beispiele guter und kooperativer Zusammenarbeit von amtlicher Statistik und privaten Datenproduzenten, in denen auch der unabdingbare Zugang zu den Metadaten gegeben ist. Hier schränken dann aber zum Teil rechtliche Rahmenbedingen und öffentliche Akzeptanz, wie derzeit in den Niederlanden,^36^ Weiterentwicklungsmöglichkeiten ein.

Die beschriebenen Entwicklungen haben erhebliche Auswirkungen auf die Qualität der Daten.^37^ Selektivitäten bei Mobilfunkdaten oder auch bei Daten aus dem Internet sind nur dann qualitätsbeschränkend, wenn sie nicht erkennbar sind. Bei qualitativ hochwertigen Befragungsdaten und bei administrativen Daten sind Fehler in den Daten aufgrund der Zugangsmöglichkeiten auch zu den Metadaten im Grunde genommen weitgehend bekannt und können dadurch in der Analyse berücksichtigt werden. Bei neuen digitalen Daten dagegen bedarf es hier noch erheblicher Untersuchungen und Entwicklungen.

Neue digitale Daten und Methoden werden weltweit seit knapp zehn Jahren intensiv in den statistischen Ämtern untersucht. Im Rahmen des ESS sind hier insbesondere das Scheveningen Memorandum zu „Big Data and Official Statistics“ und die daraus initiierten Projekte zu nennen.^38^ Mit dem Scheveningen Memorandum beschlossen die Leiterinnen und Leiter der Statistischen Ämter im ESS, nicht länger über die Potenziale und Herausforderungen von Big Data zu diskutieren, sondern im Rahmen konkreter Projekte an der Einsatzmöglichkeit der neuen digitalen Daten für amtliche Statistikprodukte zu forschen.

Mit den Projekten ESSnet Big Data I und II wurden dann in den Jahren 2016 bis 2020 wesentliche Erkenntnisse zum Einsatz nicht-traditioneller Daten für die amtliche Statistikproduktion erarbeitet.^39^ Nachfolgende Arbeitspakete wurden im ESSnet Big Data I in wechselnder Besetzung durch die statistischen Ämter der EU, gemeinsam mit den EFTA und zum Teil mit den Kandidatenländern, umgesetzt:

- Web scraping job vacancies
- Web scraping enterprise characteristics
- Smart meters
- AIS Vessel Identification Data
- Mobile phone data
- Early estimates
- Multiple domains
- Methodology

Im Rahmen des ESSnet Big Data II wurden die Arbeiten dann in den nachfolgenden Kapiteln fortgesetzt:

- Follow up workpackages of the first implementation phase
- Workpackages for new pilot projects
- Workpackages concerning trusted smart statistics

Die Ergebnisse der beiden Projekte wurden in einer abschließenden Konferenz präsentiert. Abstracts, Folien und Vortagsvideos finden sich hierzu unter https://ec.europa.eu/eurostat/cros/content/bdes-2020_en.

Seit 2018 werden die Projektergebnisse unter dem Titel ‚Trusted Smart Statistics‘ in verschiedenen Projekten fortentwickelt.^40^ Das ESSnet ‚Web Intelligence Network‘ setzt sich dabei mit den verschiedenen Möglichkeiten der Nutzung von Daten aus dem Internet auseinander.^41^ Ganz konkret sollen z. B. Informationen aus dem Unternehmensregister der statistischen Ämter mit Daten über diese Unternehmen aus dem Internet zusammengeführt werden.^42^ Ziel ist es hier insbesondere auch, eine Plattform mit Programmanwendungen, Best Practise Beispielen und erläuternden Informationsmaterialien für das ‚Network‘ der statistischen Ämter in Europa zu schaffen.

Das ESSnet ‚Trusted Smart Surveys‘ untersucht für die amtliche Statistik die Fragestellung, wie neue digitale Datenquellen mit Befragungsdaten kombiniert werden können.^43^ Schon heute sind Befragungen häufig ‚smart‘, das heißt die Fragebögen können auf allen elektronischen Kommunikationsgeräten beantwortet werden. ‚Trusted‘ werden die Erhebungen, u. a. wenn die Auskunftgebenden darüber hinaus auch den Zugriff z. B. auf private Sensoren erlauben, so z. B. den Standortdaten des Mobilfunkgeräts oder zum Fitnessarmband ermöglichen. Erste Pilotanwendungen werden derzeit für die Statistik zur Zeitverwendung sowie für komplexe Haushaltsstatistiken, die auf der Grundlage von Haushaltsbüchern entstehen, vorbereitet.

Bislang sind neue digitale oder auch als nicht-traditionell bezeichnete Daten aber nur in ausgewählten Teilbereichen in den amtlichen Statistikproduktionsprozess integriert worden, etwa in den Preisstatistiken^44^. Wesentliche Fragen zur Qualität – sowie der Zugang zu Daten wie Metadaten – sind weiterhin noch ungeklärt. Nichts desto trotz sind schon sehr vielfältige Ergebnisse im Rahmen der internationalen und nationalen Projekte entstanden. Hier ist die Motivation der statistischen Ämter diese auch zu veröffentlichen. Die Herausforderung hierbei ist, dass die Ergebnisse Resultate der statistischen Ämter sind, aber (noch) nicht die Qualität amtlicher Statistiken aufweisen. Eine ganze Reihe von Ämtern publiziert daher mittlerweile eine Rubrik ‚Experimentelle Daten‘ im Rahmen ihrer Internetangebote. So auch das Statistische Bundesamt, das die Seiten ‚EXDAT‘ seit Anfang 2020 veröffentlicht, mittlerweile liegen hier Ergebnisse für elf Projekte vor.^45^ Insbesondere der Mobilitätsindikator, der wöchentlich aktualisiert auf der Kreisebene Mobilität auf der Grundlage von Mobilfunkdaten mit Inzidenzzahlen verbindet, hat einen großen Anklang.^46^ Leider zum Teil nicht mit der sachgerechten Einordnung seitens der Nutzer, die diese Ergebnisse teilweise als amtlich bezeichnen. Gerade aber bei den teilweise hoch selektiven Mobilfunkdaten sind diese Daten vieles, aber nicht amtlich.

### Ausblick

Für evidenzbasierte Entscheidungen standen der Politik, der Wirtschaft und der Wissenschaft noch nie so viele Daten zur Verfügung wie heute. Aber eine reine Datenfülle bildet die ‚Realität‘ noch nicht ab, wie aktuell beispielsweise ganz deutlich die Corona-Krise zeigt.^47^

Mindestens ebenso wichtig wie die Nutzbarmachung bereits existierender digitaler Daten ist für die amtliche Statistik eine intensivere Befassung mit aktuellem und künftigem Informationsbedarf. Denn für die amtliche Statistik gilt Klasse statt Masse. Die Relevanz amtlicher Daten für aktuelle politische Fragestellungen ist schlussendlich ausschlaggebend für die Wahrnehmung von Statistik durch die Politik, Wirtschaft und Gesellschaft. Trägt die Statistik – wenn auch nur indirekt durch Aufklärung – zur Einhaltung des Klimaabkommens von Paris und des Erreichens von Klimaneutralität bei? Welche Informationsbedarfe ergeben sich mit der digitalen Transformation? Wie etwa können die politischen Ziele gleichwertiger Lebensbedingungen und gesellschaftlicher Zusammenhalt adäquat statistisch abgebildet werden? Dies alles sind Fragen, mit denen sich auch die statistischen Ämter dringend befassen müssen. Unterstützung durch die Wissenschaft kann dabei hilfreich sein, Unterstützung durch die Politik wäre zwingend erforderlich. Diese sollte die amtliche Statistik von der Zwangsjacke der Inputorientierung des deutschen Statistikrechts befreien. Warum muss in Deutschland jede Frage und jedes Merkmal einer statistischen Erhebung in einem langwierigen Prozess vom Gesetzgeber genau festgelegt werden? Wenn dieser sich – wie im Übrigen in der europäischen Statistik weit verbreitet – darauf beschränken würde, qualitativ hochwertigen Output zu beschreiben und gesetzlich anzufordern, würde dies Anpassungen des Programms der Statistik an den aktuellen Bedarf massiv erleichtern und die Flexibilität des Statistiksystems erhöhen.

Um ihre Rolle als qualitativ hochwertiger Datenproduzent auch künftig angemessen ausfüllen zu können, muss sich die amtliche Statistik in diesem Umfeld erneut weiterentwickeln. Traditionell erhobene Daten werden auch künftig erforderlich sein, ebenso aber eine konsequente Einbeziehung geeigneter neuer digitaler Daten. Dabei wird es eine erhebliche Herausforderung sein, den Markenkern der amtlichen Statistik und ihre im Code of Practice formulierten Qualitätsanforderungen in die Welt der neuen digitalen Daten zu transformieren. Der ‚Lösung‘ des Adäquationsproblems kommt dabei eine ganz besondere Bedeutung zu. Gelingt dies, so kann die Einbeziehung neuer digitaler Daten in die amtliche Statistik deren Anwendungsbereiche massiv erweitern und die Akzeptanz des Instruments selbst erhöhen. Selbstverständlich können grundsätzlich auch nichtamtliche Statistiken Teile der ‚Realität‘ gut abbilden. Hinsichtlich ihrer Qualität sind sie aber für die Nutzerinnen und Nutzer oft nur schwer einschätzbar. Ein Label ‚Produziert nach dem European Code of Practice‘ vergeben durch eine ‚statistical authority‘ kann hier eine Orientierungshilfe sein. Beispielgebend könnte hier die UK Statistics Autorithy sein.^48^ Diese ist bisher nur für die amtliche Statistik zuständig, aber die grundsätzliche Idee ließe sich weiterentwickeln.

## Befreit die amtlichen Firmendaten von ihren Schützern!

*Joachim Wagner*

### Die Ausgangslage: Restriktionen beim Zugang zu amtlichen Firmendaten

Firmendaten – Daten für Unternehmen oder Betriebe – aus Erhebungen der Statistischen Ämter des Bundes und der Länder unterliegen den strengen Schutzbestimmungen des Bundesstatistikgesetzes (BStatG) und entsprechender Gesetze der Bundesländer. Einzelangaben sind geheim zu halten, sie sind für Außenstehende – und hierzu zählen auch Wissenschaftler – nur sehr eingeschränkt in faktisch anonymisierter Form bzw. in speziell abgesicherten Bereichen der Ämter (Forschungsdatenzentren) zugänglich. Wissenschaftler müssen für die Nutzung von Einzeldaten in den Forschungsdatenzentren begründete Anträge stellen. Für den Zugang zu den Mikrodaten entstehen den Nutzern Kosten, die oft eine sehr erhebliche Höhe erreichen. Die mit diesen Daten produzierten Ergebnisse werden von den Statistischen Ämtern den Wissenschaftlern erst nach – oftmals sehr zeitaufwändiger – Prüfung übermittelt, wenn durch diese Übermittlung keine Geheimschutzvorschriften verletzt werden. Die Zugangshürden zu Firmendaten aus der amtlichen Statistik sind damit generell sehr hoch. In vielen Fällen sind diese Daten für externe Wissenschaftler darüber hinaus überhaupt nicht auswertbar – dazu unten mehr.

### Konsequenzen 1: Beispiele aus dem Forschungsfeld „Internationale Firmentätigkeit“

Empirische Studien mit amtlichen Firmendaten dienen u. a. der Dokumentation von Fakten, der Überprüfung modelltheoretischer Überlegungen und der Fundierung evidenzbasierter Wirtschaftspolitik. Die Restriktionen beim Zugang zu diesen Daten be- bzw. verhindern in erheblichem Ausmaß die Arbeit von Wissenschaftlern in allen diesen Bereichen. Beispiele aus dem breiten Untersuchungsfeld der internationalen Firmentätigkeit – dem zentralen Gebiet meiner wissenschaftlichen Arbeit – sollen dies illustrieren:

Deutschland ist einer der wichtigsten Akteure auf den internationalen Märkten für Güter und Dienstleistungen, wo sowohl bei Importen als auch bei Exporten auf einem der vorderen Rangplätzen steht. Seine Bedeutung als Ursprungs- und Zielland grenzüberschreitender Investitionen ist vergleichbar hoch. International agierende Firmen sind daher von zentraler Bedeutung für die kurz-, mittel- und langfristige Dynamik der deutschen Volkswirtschaft insgesamt und ihrer Sektoren und Regionen.

Verlässliche und aktuelle Daten über die internationale Tätigkeit von Firmen in Deutschland sind vor diesem Hintergrund von hoher Relevanz. Aus der amtlichen Statistik liegen hierzu vielfältige Informationen vor, u. a. zu den grenzüberschreitenden Transaktionen von Firmen mit Sitz in Deutschland. Das Statistische Bundesamt stellt für die Berichtsjahre 2009 bis 2014 Informationen zu den grenzüberschreitenden Transaktionen von Gütern für die am Außenhandel beteiligten Firmen bereit. Diese Daten enthalten u. a. Informationen über exportierte und importierte Güter (Werte, Mengen) sowie Zielländer (für Exporte) und Ursprungsländer (für Importe) – Wer exportierte bzw. importierte welche Güter zu welchem Wert und in welcher Menge in welches Land bzw. aus welchem Land? Diese Daten, die im Forschungsdatenzentrum des Statistischen Bundesamtes zugänglich sind, sind eine sehr wertvolle Informationsquelle über den internationalen Güterhandel von Firmen in Deutschland (vgl. hierzu als Überblick Wagner (2018) und die in Wagner (2019) abgedruckten Studien).

Diese Firmendaten beruhen auf Transaktionsdaten, die als prozessproduzierte Daten beim grenzüberschreitenden Handel mit Nicht-EU-Ländern anfallen oder die auf Meldungen für die Statistik des Intra-EU-Handels beruhen. Eine entsprechende Aufbereitung dieser Transaktionsdaten zu Firmendaten für die Jahre vor 2009 und nach 2014 erfolgte nicht. Diese Restriktion beim Datenzugang verhindert damit die empirische Forschung auf zahlreichen sehr spannenden Gebieten. So ist es u. a. nicht möglich, die Konsequenzen der großen internationalen Finanzkrise von 2007/2008 für die Güterhandelsströme detailliert zu untersuchen. Ebenso können die Folgen der Handelsrestriktionen, die von der Europäischen Union seit Mitte 2014 als Reaktion auf die Annexion der Krim gegen Russland verhängt und die in der Folgezeit mehrfach verlängert wurden, für die international aktiven Firmen nicht empirisch analysiert werden. Und die Auswirkungen des aktuellen „Handelskrieges“ der USA mit China für den Außenhandel von Firmen in Deutschland lassen sich ebenfalls nicht analysieren, was für evidenzbasierte wirtschaftspolitische Beratung sehr hinderlich ist.

Neben dem internationalen Güterhandel spielen grenzüberschreitende Investitionen eine zentrale Rolle in der internationalen Firmentätigkeit. Hier sorgen insbesondere Übernahmen deutscher Firmen durch Investoren aus China seit einigen Jahren für zumeist negative Schlagzeilen (Stichwort „technologischer Ausverkauf“). Verlässliche Informationen zu Investitionen deutscher Firmen im Ausland und ausländischer Firmen in Deutschland liegen nicht bei den statistischen Ämtern des Bundes und der Länder vor, denn deren Sammlung und Aufbereitung ist Aufgabe der Deutschen Bundesbank. Für externe Wissenschaftler sind diese Direktinvestitionsdaten in der Bundesbank in deren Forschungsdatenzentrum zugänglich, aber diese Daten können nicht mit den zahlreichen Informationen zu den Firmen aus den Beständen der statistischen Ämter verknüpft werden. Daher ist es auch nicht möglich, die Auswirkungen von Übernahmen deutscher Firmen durch chinesische Firmen auf die wirtschaftliche Entwicklung der übernommenen Firmen wie z. B. deren Investitions- und Innovationsverhalten empirisch zu analysieren. Dies schränkt jede Möglichkeit einer evidenzbasierten Wirtschaftspolitik in diesem Kontext sehr stark ein (wenn sie dadurch nicht sogar ausgeschlossen wird).

Gleiches gilt für den grenzüberschreitenden Handel mit Dienstleistungen. Auch hier liegen die Informationen vor, sie sind allerdings ebenfalls nur in den Räumen der Deutschen Bundesbank zugänglich – und sie sind nicht mit den Informationen zum internationalen Güterhandel der Firmen und mit weiteren Informationen zu diesen Firmen verknüpft.

Ein weiterer sehr wichtiger Bereich internationaler Firmentätigkeit, über den aktuelle Informationen in der amtlichen Statistik vorliegen, die externen Wissenschaftlern nicht zugänglich sind, betreffen grenzüberschreitende Verlagerungen von Tätigkeiten in Deutschland ansässiger Unternehmen. Hierzu wurde für das Berichtsjahr 2016 eine Umfrage durch Statistische Ämter durchgeführt (vgl. Kaus 2019). Die Daten dieser Umfrage sind nicht in den FDZ verfügbar; das Analysepotenzial dieser Daten, das durch eine Verknüpfung mit vorhandenen weiteren Informationen zu verlagernden und nicht verlagernden Firmen deutlich gesteigert werden könnte^49^, wird daher kaum genutzt.

Diese Restriktionen beim Zugang zu den Firmendaten und bei ihrer Verknüpfung über die Grenzen der Datenproduzenten (Statistische Ämter, Bundesbank) hinweg verhindern damit fundierte empirische Analysen in zentralen Bereichen der deutschen Volkswirtschaft!^50^

### Konsequenzen 2: Restriktiver Datenzugang verhindert Reproduzierbarkeit in der empirischen Forschung und verschärft die Replikationskrise

Der restriktive Zugang zu den Firmendaten aus der amtlichen Statistik, der durch hohe Hürden bei der Antragstellung, bei der Wartezeit auf die geprüften Ergebnisse und insbesondere bei den Kosten der Datennutzung (die ja zudem wegen der vertraglichen Regelungen in den Forschungsdatenzentren alle drei Jahre erneut anfallen!) gekennzeichnet ist, vermindert nicht nur die Anreize für eine Nutzung dieser Daten durch Wissenschaftler (insbesondere durch Nachwuchswissenschaftler mit befristeten Verträgen und entsprechend hohem Zeitdruck bei der Anfertigung von Qualifikationsarbeiten) und bremst damit den wissenschaftlichen Fortschritt in vielen wichtigen Feldern. Der restriktive Datenzugang verhindert zudem eine Reproduktion von empirischen Ergebnissen, die mit diesen Daten erstellt wurden, durch andere Wissenschaftler:

Anders als in vielen Fällen heute üblich können die in einer ökonometrischen Studie verwendeten Firmendaten aus der amtlichen Statistik ja bei einer Publikation nicht zusammen mit dem verwendeten Code und den Ergebnisfiles in einem Datenarchiv des Journals oder einem Repositorium abgelegt und dann von interessierten Wissenschaftlern für Replikationsstudien (Überprüfung der publizierten Ergebnisse, Tests auf deren Robustheit bei Verwendung anderer Methoden oder Modellspezifikationen etc.) einfach genutzt werden. Prinzipiell ist es selbstverständlich möglich, dass an Replikationen interessierte Dritte den Code der Primärforscher nutzen und in den Forschungsdatenzentren der Statistischen Ämter diese Studien replizieren – aber die Hürden der Datennutzung sind dabei genauso hoch wie für die Primärforscher. Angesichts der deutlich geringeren Anreize für Replikationsstudien in Form von geringeren (hochkarätigen) Publikationsmöglichkeiten und damit geringeren Beiträgen zur wissenschaftlichen Reputation verwundert es nicht, dass solche Replikationsstudien von Beiträgen auf der Basis von amtlichen Firmendaten unterbleiben.^51^

Der restriktive Zugang zu den Firmendaten aus der amtlichen Statistik verhindert damit die Reproduzierbarkeit empirischer Forschung mit diesen Daten. Dies hat weit reichende Folgen, die McCullough und Vinod (2002, S. 888) schon vor vielen Jahren drastisch so beschrieben haben:

Replication is the cornerstone of science. Research that cannot be replicated is not science, and cannot be trusted either as part of the profession’s accumulated body of knowledge or as a basis for policy … A researcher who does not openly allow independent verification of his results puts these results in the same class as the results of a researcher who does share his data and code but whose results cannot be replicated: the class of results that cannot be verified, i.e. the class of results that cannot be trusted.

Die Schuld liegt allerdings in der hier betrachteten Situation nicht bei den Forschern, die die Originalstudien erstellt haben (es sei denn, sie verweigern interessierten Dritten den Zugang zu dem von ihnen für ihre Originalstudien verwendeten Code), sondern bei institutionellen Regelungen, die den Zugang zu den Daten der Originalstudien derart restriktiv gestalten.

Der restriktive Datenzugang verschärft die zu Recht vielfach beklagte Replikationskrise, da dadurch die Kosten für die Durchführung von Replikationen auf der Basis von amtlichen Firmendaten enorm gesteigert werden.

### Es gibt Lösungen!

Einzeldaten aus Erhebungen der amtlichen Statistik in Firmen sind streng vertraulich, und hierfür gibt es gute Gründe. Die Firmen sind zur wahrheitsgemäßen Auskunft verpflichtet und müssen Informationen mitteilen, die sie in sehr vielen Fällen als Geschäftsgeheimnis betrachten. Wer möchte schon gerne, dass seine Konkurrenten, Lieferanten oder Kunden Einzelheiten z. B. über die Kostenstruktur oder die detaillierte Absatzentwicklung erfahren? Informationen, die einzelnen Firmen zugeordnet werden können, dürfen daher von den statistischen Ämtern nicht veröffentlicht werden. Diese Informationen dürfen auch nicht an Wissenschaftler weitergegeben werden, auch wenn diese gar kein wirtschaftliches Interesse an diesen Angaben haben sondern mit ihnen wissenschaftliche Analysen durchführen möchten, für die die Kenntnis der Identität des Merkmalsträger nicht relevant ist.

Bis vor 25 Jahren bedeutete dies, dass Wissenschaftler die wirtschaftsstatistischen Datenschätze in den statistischen Ämtern über die publizierten Tabellenbände hinaus nur in Form von Sonderauswertungen nutzen konnten, in denen Mitarbeiter der Ämter nach Vorgaben der Forscher spezifische zusätzliche Auswertungstabellen erstellten. Eigene mikroökonometrische Analysen der Wissenschaftler mit den vertraulichen Firmendaten waren nicht möglich. Für uns stand über der Tür der statistischen Ämter der Spruch, der bei Dante Alighieri seit über 700 Jahren in *La divina commedia *als Inschrift über dem Eingang der Hölle steht:

Lasciate ogne speranza, voi ch’intrate.^52^

Mit der Implementierung der Forschungsdatenzentren (FDZ) im Statistischen Bundesamt bzw. in den statistischen Ämtern der Länder ab 2001 hat sich diese Situation grundlegend geändert.^53^ Es ist unstrittig und durch zahlreiche nationale und internationale wissenschaftliche Publikationen belegt, dass die in den FDZ der statistischen Ämter verfügbaren Firmendaten eine Basis für innovative deskriptive Analysen und qualitativ hochwertige mikroökonometrische Forschung bilden. Die wissenschaftliche Nutzung dieser Daten bringt nicht nur Erträge für die Wissenschaftler selbst, sie trägt auch zu einem besseren Verständnis realer ökonomischer Strukturen und Prozesse bei, und sie kann zu einer besseren evidenzbasierten Wirtschaftspolitik beitragen. Hierbei treten jedoch bei allem Fortschritt bezüglich des Zugangs von Wissenschaftlern zu den Datens(ch)ätzen der amtlichen Statistik immer noch und teils heute sogar verstärkt Probleme auf, die mit den beiden Stichworten „Zeit“ und „Geld“ gekennzeichnet werden können:

#### Zeit

erfordert die Arbeit mit diesen Firmendaten nicht nur bei der Ausarbeitung des Nutzungsantrags, beim Warten auf die Genehmigung des Antrags durch alle statistischen Landesämter und beim Warten auf die Bereitstellung der Daten nach der Aufbereitung im FDZ. *Zeit* – aus der Sicht der Nutzer immer sehr viel Zeit und oft viel zu viel Zeit – nimmt insbesondere die Prüfung der erzeugten Ergebnisse auf Geheimhaltungsfreiheit durch die Mitarbeiter im FDZ in Anspruch. Auch wenn ein Austesten von Programmen mit „sinnfreien“ Testdatensätzen am eigenen Rechner vielfach möglich ist – bis zu einer ersten Fassung eines Papers sind in der Regel zahlreiche Fehlversuche zu verzeichnen und viele Läufe mit Modellvarianten erforderlich. Jede Runde kostet dabei in der Regel mehrere Tage, viel zu oft auch Wochen. Für Forscher, die gewohnt sind mit Mikrodaten auf dem eigenen PC zu arbeiten, ist die zeitaufwendige kontrollierte Datenfernverarbeitung im FDZ in der Regel äußerst gewöhnungsbedürftig. Nicht wenige potenzielle Nutzer – darunter viele Nachwuchswissenschaftler mit oft kurz laufenden Zeitverträgen – entscheiden sich auch deshalb gegen die Arbeit mit diesen Daten der amtlichen Statistik.

#### Geld

erfordert die Arbeit mit den Firmenpaneldaten in den FDZ, weil für die Nutzung der Daten Gebühren erhoben werden. Man kann leicht ausrechnen, dass ein kombinierter Firmenpaneldatensatz, der aus den Daten mehrerer Erhebungen zusammengestellt wird, sehr schnell sehr teuer wird. Hinzu kommt dann noch das Entgelt für die projektspezifische Aufbereitung der Daten.^54^ Und das alles muss alle drei Jahre neu bezahlt werden, da Projekte nur maximal drei Jahre laufen dürfen und die Datennutzung dann neu zu beantragen und neu zu bezahlen ist. Ein ökonomisch rational handelnder potenzieller Datennutzer wird Kosten und Nutzen einer Verwendung der FDZ-Daten abwägen. Hohe Kosten fallen durch den erforderlichen Aufwand an Zeit und Geld mit Sicherheit an. Der Nutzen ist potenziell hoch, aber unsicher, da der Ertrag jedes ergebnisoffenen Forschungsvorhabens Null oder sehr gering sein kann. Daher wird eine Entscheidung oft gegen eine Nutzung der Firmenpaneldaten ausfallen. Ich finde das sehr bedauerlich.

Zugegeben, die Möglichkeiten zur Arbeit mit vertraulichen wirtschaftsstatistischen Einzeldaten aus Erhebungen der amtlichen Statistik haben sich für externe Wissenschaftler in den vergangenen 25 Jahren in einem Ausmaß verbessert, das wir zu Beginn nicht für möglich gehalten haben. Aber dabei dürfen wir nicht stehen bleiben.

Erforderlich ist erstens eine Bereitstellung der ja bereits vorhandenen und mit öffentlichen Mitteln finanzierten Daten zu Grenzkosten-Preisen (aus volkswirtschaftlicher Sicht eine selbstverständliche Forderung, die aber nach meiner Erfahrung schwer zu vermitteln ist), wobei für die Finanzierung eine Lösung zu finden wäre, die durch Zahlung einer „Flatrate“ einer Förderinstitution wie der Deutschen Forschungsgemeinschaft oder durch eine Zuwendung des für Forschung zuständigen Bundesministeriums die Wissenschaftler von der mühsamen Arbeit der (Dritt)Mittelbeschaffung befreien würde.

Zweitens muss der Aufwand für die zeit- und personalintensive (und damit für alle Beteiligten sehr teure) Geheimschutzprüfung der Ergebnisse sehr weitgehend reduziert werden. Hierfür gibt es unterschiedliche Lösungen:

- Ein sehr vielversprechender Ansatz hierzu ist eine technische Lösung in Form des Systems *JoSuA *(Job Submission Application), das bereits im FDZ des Instituts für Arbeitsmarkt- und Berufsforschung der Bundesagentur für Arbeit eingesetzt wird. Ohne hier auf Einzelheiten eingehen zu können^55^ ist der Kerngedanke dieser technischen Lösung der, dass Nutzer von ihrem Arbeitsplatz aus Berechnungen mit den vertraulichen Einzeldaten durchführen können und die Ergebnisse dann „sehen“ – aber nicht „ausdrucken“ dürfen. Erst wenn die empirischen Analysen aus der Sicht der Nutzer „fertig“ sind, erfolgt eine endgültige Geheimschutzprüfung und Freigabe der Ergebnisse, die dann dem Wissenschaftler zur weiteren Verwendung übermittelt werden. Dieser erprobte Weg der Arbeit mit vertraulichen Einzeldaten wird bereits genutzt – allerdings nicht bei der Arbeit mit Firmendaten aus den Statistischen Ämtern oder der Deutschen Bundesbank.
- Ein alternativer Weg, der vor kurzem von Ulli Rendtel vorgeschlagen wurde, sieht einen radikalen Paradigmenwechsel beim Zugang der Wissenschaft zu Daten der amtlichen Statistik vor, bei dem das Kontrollprinzip durch das Vertrauensprinzip ersetzt wird (vgl. Rendtel 2014). Aus meiner Sicht sind dies sehr bedenkenswerte Überlegungen, wobei ich die Chancen für eine Umsetzung allerdings leider eher als sehr gering einschätze.

Das Ziel, amtliche Firmendaten von ihrem Schützern zu befreien und in den Forschungsdatenzentren für Wissenschaftler kostenlos an 365 Tagen im Jahr 24 h im Remote-Access verfügbar zu machen, wird daher trotz der Verfügbarkeit gesetzeskonformer technischer Lösungen noch lange ganz oben auf der Agenda von empirisch arbeitenden Wirtschaftswissenschaftlern stehen.

## (K)ein Platz für Survey-Statistik? Der Masterstudiengang Survey-Statistik an der Otto-Friedrich-Universität Bamberg

*Florian Meinfelder*

### Ein neues Masterprogramm an der Universität Bamberg

Zum Wintersemester 2010/11 führte meine leider viel zu früh verstorbene ‚Doktormutter‘ Susanne Rässler den Masterstudiengang Survey-Statistik ein – mit einer Empfehlung des RatSWD im Rücken und trotz einiger Bedenken von Fakultätsseite, denn schließlich hing das Programm zum allergrößten Teil an ihrem eigenen Lehrstuhl für Statistik und Ökonometrie. Ein wichtiges Argument für die Genehmigung war letztendlich die Kooperation mit der FU Berlin und der Universität Trier^56^, mit denen man im Rahmen eines sogenannten ‚Teleteaching‘-Programms Veranstaltungen teilte und dies auch heute noch tut. Dies ist in meinen Augen generell ein probates Mittel, Masterprogramme zu initiieren, wenn die eigene personelle Ausstattung allein dies nicht ermöglicht. Im Übrigen hat der Corona-bedingte Lockdown im Frühjahr 2020 Online-Lehre etwas hoffähiger gemacht.

### Die Ausgangssituation

Wie so viele Lehrstühle für Statistik und/oder Ökonometrie an WISO- oder SOWI-Fakultäten anderer Universitäten sind wir Einzelkämpfer – an größeren Fakultäten gibt es manchmal auch einen zweiten Lehrstuhl. Wir sind an unserer Fakultät keiner Fachgruppe zugehörig, und im Organigramm werden wir unter ‚Weitere Fächer‘ geführt, aber uns obliegt die komplette Bachelor-Statistik-Ausbildung für zwei Fakultäten (neben Studierenden der eigenen SOWI-Fakultät haben auch Studierende der Wirtschafts- und Angewandten Informatik das Vergnügen mit uns).

Fast alle wirtschafts- und sozialwissenschaftlichen (bzw. rechts- und wirtschaftswissenschaftlichen) Fakultäten an deutschen Universitäten haben bei der Besetzung ihrer Lehrstühle finanzstatistische beziehungsweise makro-ökonometrische Forschungsschwerpunkte gesetzt und hierdurch (unfreiwillig) zu einer gewissen ‚Monokultur‘ in der statistischen Forschungslandschaft geführt. Auch wenn der Bamberger Lehrstuhl ‚Lehrstuhl für Statistik und *Ökonometrie*‘ heißt, hat Susanne Rässlers Lehr- und Forschungsschwerpunkt zu einer etwas anderen (breiteren?) Ausrichtung geführt, von der die traditionell empirisch ausgerichtete Bamberger Soziologie sowie Politologie und vor allem das noch junge Leibniz-Institut für Empirische Bildungsverläufe (LIfBi) profitierten.

### Ausrichtung und Tätigkeitsspektrum der Absolventen

Der Master (in) Survey Statistik (abgekürzt durch das Akronym ‚MiSS‘, welches zugleich auf den Forschungsschwerpunkt der Analyse unvollständiger Daten verweist) an der Universität Bamberg ist ein Studium, dessen Pflichtprogramm Akzente in der amtlichen und in der Survey-Statistik, aber auch in der mathematischen Statistik oder der Survey-Methodik setzt. Neu-MiSSianer haben häufig einen Bachelor-Abschluss in einem sozialwissenschaftlichen oder statistischen Studiengang. Lingua Franca ist die (tatsächlich freie) Programmiersprache *R*.

Ursprünglich war das Lehrangebot des MiSS darauf ausgerichtet, Leute primär für den Bereich der amtlichen Statistik auszubilden, und seit 2016 können Studierende zusätzlich das EMOS-Zertifikat (European Master in Official Statistics) erwerben. Der Masterstudiengang hat sich inhaltlich mittlerweile zu einem vielseitigen Programm im Bereich der angewandten Statistik mit survey-statistischem Schwerpunkt gewandelt, so dass unsere Absolventen sehr vielseitig – jedoch nie fachfremd – in den Arbeitsmarkt^57^ einsteigen. Grob lässt sich dies in folgende Tätigkeitsfelder (mit exemplarischen Arbeitgebern^58^ in Klammern) unterteilen:

- Automotive (Daimler AG: eine laufende externe Promotion und vier externe Masterarbeiten in der F&E-Abteilung in Ulm, MAN SE: zwei externe Masterarbeiten)
- Healthcare (Boehringer Ingelheim AG, Novartis AG/GlaxoSmithKline plc., Siemens Healthineers AG)
- Markforschung (GfK SE: eine extern abgeschlossene Promotion und fünf externe Masterarbeiten, Kantar TNS/Kantar Added Value, OPINION Market Research & Consulting GmbH)
- E‑Commerce (externe Masterarbeiten bei Baur Versand GmbH & Co. KG, Otto GmbH & Co KG, Witt Gruppe)
- Finanzbereich und Versicherungen (Deutsche Bundesbank, HUK-COBURG: zwei externe Masterarbeiten)
- Amtliche Statistik (Destatis, Institut für Arbeitsmarkt- und Berufsforschung, Bayerisches Landesamt für Statistik)
- Academia (Otto-Friedrich-Universität Bamberg: Laufende und abgeschlossene Promotionen am Lehrstuhl für Statistik und Ökonometrie, sowie dem LIfBi, der BAGSS und anderen Lehrstühlen der Fakultät SOWI, Friedrich-Alexander-Universität Erlangen-Nürnberg: zwei laufende Promotionen und diverse Masterarbeiten am Institut für Medizininformatik, Biometrie und Epidemiologie, Universität Trier: eine laufende Promotion und eine externe Masterarbeit, GESIS: Eine laufende externe Promotion, FU Berlin und TU Darmstadt: je eine externe Masterarbeit)
- Sonstige (Nintendo of Europe GmbH: externe Masterarbeit, diverse Statistik- und Data Science-orientierte Start-Ups und Consulting-Firmen)

Grundsätzlich sind die Perspektiven am Arbeitsmarkt für unsere Absolventen sehr gut, da viele Firmen datenbasierte Informationsgewinnung forcieren, was sich in neugegründeten Abteilungen niederschlägt, die Namen wie *Business Intelligence* oder *Predictive Analytics* tragen. Auf Grund der Wahlmöglichkeiten im Bereich der computergestützten Datenaufbereitung und -analyse und der vermittelten Programmierkenntnisse sind Bamberger Survey-Statistiker darüber hinaus für das noch relativ neue Berufsbild *Data Science* attraktiv.

### Die Entwicklung in Zahlen

Der Masterstudiengang hat seit den Anfangstagen vor zehn Jahren eine erstaunliche Entwicklung genommen und ist eine Erfolgsgeschichte an der Universität Bamberg geworden.

Die folgenden abschließenden Zählungen zeigen die Entwicklung der Studierendenzahlen seit dem Wintersemester 2010/11. Zum Zeitpunkt der Entstehung dieses Textes sind 107 MiSS-Studierende eingeschrieben (Stand: 02.11.2020). Von den 29 Neuanfängern haben die große Mehrheit ihren Bachelorabschluss nicht an der Otto-Friedrich-Universität Bamberg erworben, so dass das Programm nicht in direkter Konkurrenz zu anderen Bamberger Angeboten steht^59^. Dieser Anteil deckte sich mit den Vorsemestern, wobei tendenziell der Anteil an Masterstudierenden von anderen Hochschulen sogar zunimmt.

**Figure 7:**
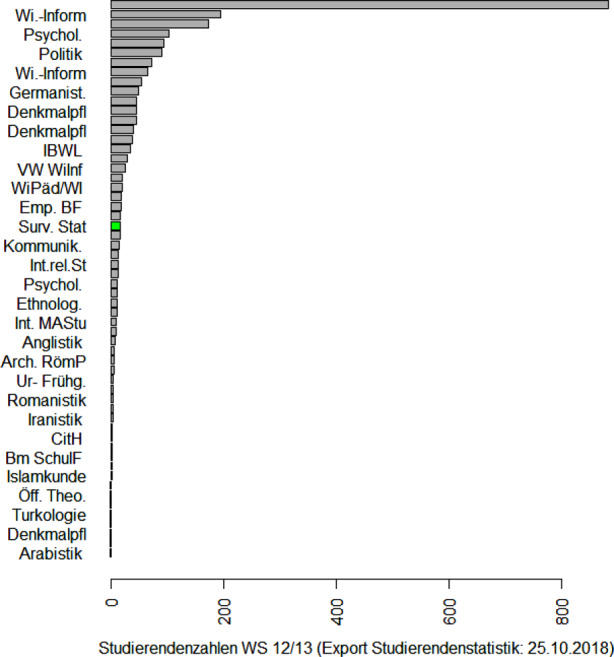

Zu diesem Zeitpunkt war der Studiengang etabliert und wurde im darauf folgenden Semester akkreditiert (Abb. 7).^60^

**Figure 8:**
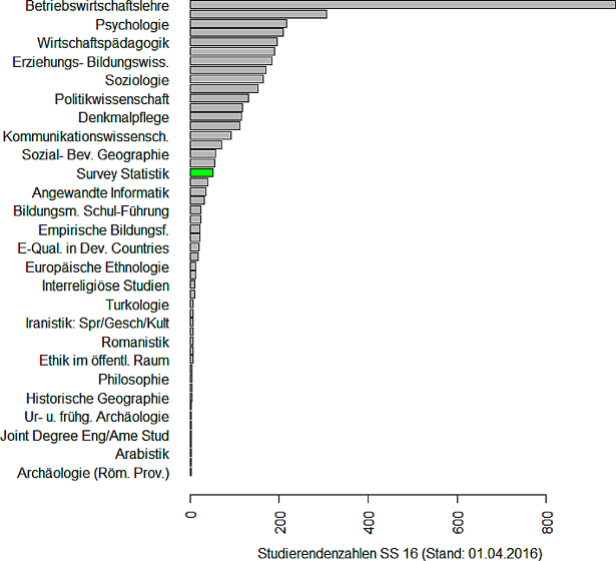

Im Sommersemester 2016 waren im Masterstudiengang Survey-Statistik bereits 52 Studierende eingeschrieben. Zu diesem Zeitpunkt wurde bereits die Anzahl der Studierenden übertroffen, für die der Studiengang ursprünglich ausgelegt war (Abb. 8).

**Figure 9:**
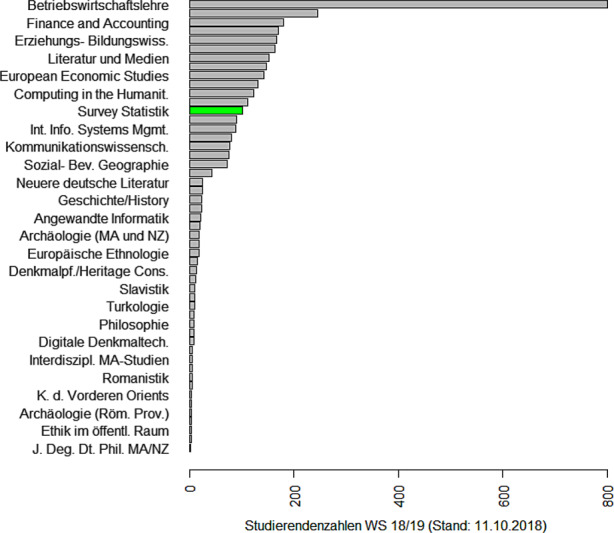

Diese Zahl hat sich in den darauf folgenden zwei Jahren nochmals verdoppelt und der ‚MiSS‘ gehört mittlerweile gemessen an der Anzahl der Studierenden zum oberen Terzil an der Otto-Friedrich-Universität Bamberg (Abb. 9).

**Figure 10:**
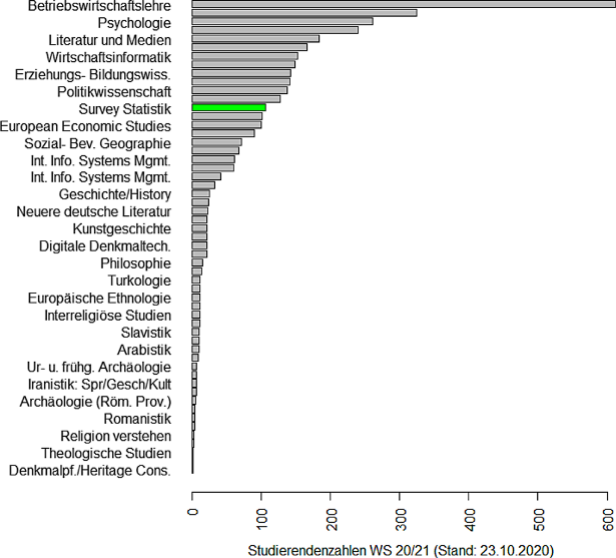

Im Wintersemester 2020/21 waren an der Otto-Friedrich-Universität Bamberg erstmals mehr Survey-Statistiker als Volkswirte („European Economic Studies“) im Master eingeschrieben (Abb. 10).

**Figure 11:**
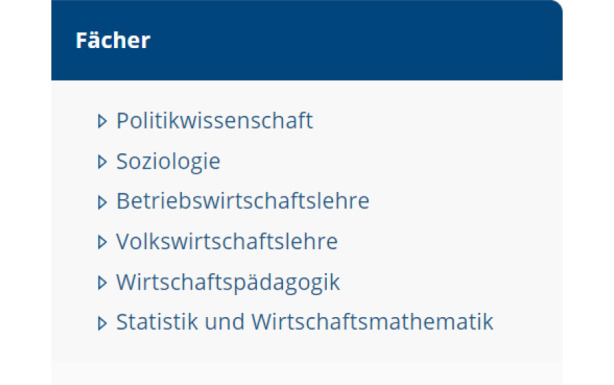

Noch immer wird ein Großteil der Lehre im MiSS durch den Lehrstuhl für Statistik und Ökonometrie gestemmt, aber seit dem Wintersemester 2019/20 gibt es Unterstützung durch die neu geschaffene Professur für Wirtschaftsmathematik und den Lehrstuhl für Survey-Statistik und Datenanalyse, eine am Leibniz-Institut für Empirische Bildungsverläufe (LIfBi) angesiedelte S‑Professur^61^. Im Organigramm der Fakultät Sozial- und Wirtschaftswissenschaften und auf der Homepage ist seitdem folgendes zu finden (Abb. 11).

Wir sind nun eine Fachgruppe! Kleine Schritte, um *Make Statistics Great Again* wahr werden zu lassen …

## Kompetenzen in der Data Science: Versuch einer Klärung anhand von Stellungnahmen der Gesellschaft für Informatik, Deutsche Statistische Gesellschaft und der DAGStat

*Ulrich Rendtel*

„Data Science“ ist ein aktueller Hype, der irgendwo zwischen Informatik und Statistik angesiedelt ist. Um die Festlegung, welche Kompetenzen ein Datenwissenschaftler haben sollte, ist in programmatischen Stellungnahmen der Gesellschaft für Informatik (GI), der Deutschen Statistischen Gesellschaft (DStatG) sowie der DAGStAT gestritten worden. Dabei kann für Statistiker ein Blick auf das von Informatikern geforderte Statistik-Profil durchaus ernüchternd sein.

Dieser Weg wird hier exemplarisch anhand der Darstellung der Kompetenzziele für ein Studium der Data Science anhand eines Weißbuchs der GI beschritten. Dabei handelte es sich um einen Entwurf, der von der DStatG und der DAGStAT kritisiert wurde. Die Auseinandersetzung mit dem GI-Entwurf führte zu einer Formulierung der Position der Statistik innerhalb der Data Science aus der Sicht der Statistiker, die hier ebenfalls dokumentiert werden soll. Erfreulicherweise führte die Auseinandersetzung dieser Fachgesellschaften zu einer teilweisen Revision des ursprünglichen GI-Entwurfs.

Data Science ist an den Informatik Fachbereichen eng verknüpft mit Begriffen wie Maschinelles Lernen^62^, Deep Learning als Modewort für Neuronale Netze, AI als Akronym für Artificial Intelligence, Knowledge Engineering oder Big Data. Diese Modeworte werden in der Regel der Informatik zugeordnet und auch von ihr in Anspruch genommen.

Diese Zuordnung kommt nicht von ungefähr. Zunächst einmal ist die Wissenschaftsgemeinde der Informatiker zahlenmäßig um Dimensionen größer als die der Statistiker. So verweist die Gesellschaft für Informatik (GI) auf 20.000 Mitglieder^63^. Dies sind fast 30 mal mehr Mitglieder als in der Deutschen Statistischen Gesellschaft. Allein aufgrund dieser Proportionen hat eine Stellungnahme der GI zur Schlüsseldisziplin Data Science mehr Gewicht als eine entsprechende Stellungnahme der DStatG.

Dieses Größenverhältnis zeigt sich beispielsweise auch im Berliner universitären Wissenschaftsbetrieb. An den drei Berliner Universitäten HU, TU und FU sind an den dortigen Instituten für Informatik jeweils 23 Professuren, also insgesamt 69 Informatik-Professuren, aufgeführt. Die Statistik-Professuren Berlins sind weit über verschiedene Fächer (Ökonomie, Mathematik, Psychologie, Veterinärmedizin) verstreut. An der FU und HU sind es jeweils 4. An der TU ist es nur eine. Zusammen mit der Biometrie-Professur an der Charite kommt man auf insgesamt 10 Berliner Statistik-Professuren.

Die Förderung der KI als Schlüsseltechnologie hat sich die Bundesregierung mit der Schaffung von 100 Professuren auf die Fahnen geschrieben^64^. Diese Professuren sind alle in der Informatik angesiedelt. Die Statistik bleibt dabei unerwähnt^65^.

Nun könnte man meinen, das Label – also die Benennung einer Professur – sei doch nebensächlich, wenn in dem Ausbildungsprogramm gediegene statistische Kompetenz vermittelt wird. Aber einige grobe Indikatoren lassen einen doch zweifeln. So ist beispielsweise in dem an der FU neu aufgelegten Master Programm Data Science^66^ die Statistik über das Modul „Statistics for Data Science“ mit gerade mal 4 Semesterwochenstunden vertreten. Aus diesem Grund lohnt es sich, sich mit den Kompetenzzielen der Informatiker für die Datenwissenschaft zu befassen.

### Kompetenzziele der Gesellschaft für Informatik für Data Science Studiengänge

Die Gesellschaft für Informatik hat sich mit den neuen Data Science Studiengängen und ihren Ausbildungszielen in einer speziellen Arbeitsgruppe beschäftigt und dazu ein White Paper herausgegeben, vgl. Gesellschaft f. Informatik (2019). Hierin wird zunächst der Begriff Data Science definiert. Diese bestehe aus „Data Engineering, Data Analytics, Data Prediction und Maschinellem Lernen“ (S. 5). In Abgrenzung zur Data Science werden verschiedene Literacies, also Fähigkeiten, beschrieben (S. 9): Data Literacy, Information Literacy, Data Information Literacy, Science Data Literacy, Digital Literacy und schließlich Statistical Literacy. Nach dem Verständnis der GI bezeichnet Statistical Literacy „die Fähigkeit, auszuwählen, was gezählt bzw. gemessen wird, wie eine zusammenfassende Statistik erzeugt wird, welche Vergleiche damit angestellt werden dürfen und wie die Ergebnisse kommuniziert werden sollen.“ Das ist in meiner Interpretation als Statistiker ungefähr die Beachtung des Messniveaus sowie die Berechnung von Mittelwerten, Standardabweichungen und die Darstellung über Histogramme.

**Figure 12:**
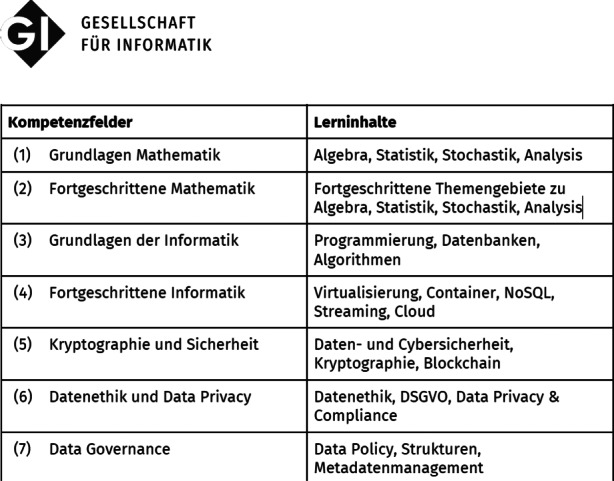

Nach der Klärung der Begriffe erfolgt die Festlegung der Kompetenzfelder, die in den Data Science Studiengängen erworben werden sollen. Insgesamt werden 14 Kompetenzfelder beschrieben (S. 18). Interessant ist die Aufzählung in der vorletzten Fassung des White Papers: Fortgeschrittene Mathematik (1&2) und Informatik (3&4), Kryptographie und Sicherheit (5), Datenethik und Data Privacy (6), Data Governance (7), Datenintegration (8), Datenvisualisierung (9), Data Mining (10), Maschinelles Lernen (11), Business Intelligence (12), Domänenspezifische Anwendungen (13) und die Implementierung von Data Science in der Organisation (14). Und wo bleibt die Statistik? Die ist unter Mathematik zu suchen, wie der folgende Screenshot zeigt (Abb. 12).

Statistik, ein mathematisches Teilgebiet zwischen Algebra und Analysis! Das ist die Quittung für eine zu formelhafte und abgehobene Darstellung der Statistik! Es ist die Wahrnehmung unseres Fachs in der Sichtweise der Autoren dieser Arbeitsgruppe. Erst auf Intervention der DStatG wurde das Statistik-Bild etwas zurechtgerückt und so heißt es in der Schlussfassung jetzt (Tab. 3).

**Table 3:** 

Kompetenzfelder	Pflicht‑/Wahlinhalte
(1) Grundlagen Mathematik & Statistik	Algebra, Stochastik, Analysis & Statistik (Deskriptive Statistik, Statistische Inferenz, Lineare Modelle, Simulation und Resampling)
(2) Fortgeschrittene Mathematik &Statistik	Fortgeschrittene Algebra, Stochastik, Analysis & Stochastik (Nichtparametrische, Multivariate, Hochdimensionale, Bayesianische, Räumliche)
(3) Grundlagen der Informatik	Programmierung, Datenbanken, Algorithmen
(4) Fortgeschrittene Informatik	Virtualisierung, Container, NoSQL, Streaming, Cloud
(5) Kryptographie und Sicherheit	Daten- und Cybersicherheit, Kryptographie, Blockchain
(6) Datenethik und Data Privacy	Datenethik, DSGVO, Data Privacy & Compliance
(7) Data Governance	Data Policy, Strukturen, Metadatenmanagement

### Die Rolle der Statistik in der Datenwissenschaft aus der Sicht der Statistik

Die pure Nicht-Erwähnung der Statistik^67^ in dem Entwurf der GI für ein Curriculum in Data Science hat in der DStatG zu einer lebhaften Diskussion über die Rolle und die Position der Statistik in der Data Science geführt.

Es reicht eben nicht nur sehr viele Daten zu haben und sich auf die Sprechweise „n = all“ zu verlassen. Man muss auch wissen, wie die Daten entstanden sind, wie sie erhoben wurden und im Zweifelsfall sollte man auch planen können, wie man die Daten oder das Experiment, das die Daten liefert, strukturiert. Und wer Vorhersagen machen will, den interessiert es meistens auch, welches Merkmal einen Einfluss auf die Vorhersage hat. Reine Black-Box Vorhersagen mögen zwar zuverlässig sein, aber man hätte doch gerne eine Begründung für seine Entscheidung. All dies sind originäre Felder der Statistik, die eine rein datengetriebene Algorithmik nicht bedienen kann.

Die hier dokumentierte Stellungnahme^68^ des Vorstands der DStatG versucht das Verhältnis zu unseren fachlichen Nachbarn zu klären. Das folgende DAGStat-Positionspapier^69^ benennt detailliert die originären Stärken der Statistik im KI-Bereich.

#### Die Rolle der Statistik für Big Data, Data Literacy, Machine Learning, KI, Analytics und Data Science – Warum die digitalisierte Informations- und Wissensgesellschaft statistische Kompetenzen braucht

*Vorstand DStatG (Dezember* *2019)*

Daten und die stark wachsende Nachfrage, diese zu verstehen und aus ihnen Informationen, Wissen und Erkenntnis zu extrahieren, sind heutzutage allgegenwärtiger als je zuvor. Die hier von Gesellschaft, Wirtschaft und Politik wahrgenommenen – und zum Teil realen – Probleme und Defizite hinsichtlich der Verfügbarkeit von Analysekompetenz werden als kritische Faktoren eingestuft. Probleme und Lösungsansätze werden unter den Begriffen *Data Literacy, Statistical Literacy, Data and Business Analytics, Künstliche Intelligenz (KI), Data Engineering, Knowledge Engineering *und *Data Science *diskutiert und haben im Wissenschaftssystem bereits zu umfangreichen Aktivitäten und Initiativen unter diesen Schlagworten geführt, von 100 zusätzlichen KI-Professuren über neue Forschungsstrukturen bis hin zu neuen Qualifizierungsmöglichkeiten und Studiengängen, letztere oftmals mit ausgeprägtem online- und E‑Learning Bezug.

Dieser Prozess, der stark von Akteuren der Nachfrageseite bestimmt wird, die oftmals keine einschlägige statistische Fachausbildung besitzen, wirft Fragen hinsichtlich der Rolle und Bedeutung der Statistik auf, insbesondere im Verhältnis zu *Data Science*.

Die *Datenwissenschaften *(*Data Science*) formen sich international als wissenschaftliches Gebiet, interdisziplinär und getrieben durch Anwendungen in der Wirtschaft, großen Teilen der Wissenschaften und der amtlichen Statistik. Anliegen sind die Erforschung und Anwendung von wissenschaftlichen Methodiken für die Informations- und Erkenntnisgewinnung aus Daten und datengesteuerte Problemlösungen durch Verarbeitung, Aufbereitung, Analyse und Inferenz von sehr großen, hochdimensionalen Datenbeständen (*Big Data, neue digitale Daten*). Das Gebiet der *Künstlichen Intelligenz *verfolgt dies speziell mit dem Ziel, intelligentes Verhalten aus Daten für selbstoptimierende KI-Systeme zu extrahieren, vor allem mit Methoden des *Machine Learnings *und *Deep Learning Networks*. Dies erfordert Kompetenzen, die in der Vergangenheit oft nur verteilt über die Fächer Informatik, Statistik und Mathematik vorlagen.

Auch wenn das Verständnis von *Data Science *aus dieser Problemlage entstanden und noch im Fluss ist, so werden doch stets Modelle, Methoden und Erkenntnisse aus der Statistik und Informatik, sowie Optimierung, Numerik und dem Anwendungsgebiet (*Domain Knowledge*) studiert, eingesetzt und problembezogen weiterentwickelt. Zwar können wichtige Spezialprobleme in den einzelnen Disziplinen sinnvoll eigenständig beforscht werden und haben diese bereits substantiell und nachhaltig befruchtet und in Teilen auch neu geformt. Dennoch ist *Data Science *als wissenschaftliches Thema durch interdisziplinäres Denken geprägt und bezieht hieraus sein besonderes Profil.

Die Deutsche Statistische Gesellschaft hat diesen Entwicklungen, die auch eine Entwicklung der Statistik waren und sind, Rechnung getragen und *Data Science *neben *Computational Statistics *und *Statistical Literacy *in ihre Fachsystematik und Jahrestagungen fest integriert. Die Entwicklung und Einordnung von *Data Science *und die besondere Rolle der Statistik wurden in Ausschüssen der Gesellschaft und Jahrestagungen ebenso diskutiert wie Literacy-Fragestellungen.

Die Statistik nimmt eine zentrale Position in den Datenwissenschaften ein. Sie ist die Wissenschaft und praktische Disziplin, die die Lösung des ersten *Big Data*-Problems der Menschheit, der Volkszählungen und Bevölkerungsstatistik, ermöglichte. Sie erforscht basierend auf der Wahrscheinlichkeitsrechnung seit jeher essentielle Kernfragen für Datenverständnis und Wissensextraktion, nämlich Datendeskription, Datenexploration und Datenanalyse sowie Stichprobentheorie und Inferenzstatistik. Theoretische Grundlagen wurden bereits vor Beginn des Computerzeitalters gelegt und praktisch angewendet. Die Statistik hat nicht nur wesentliche Grundlagen einer theoretischen Fundierung vieler Verfahren des *Machine Learnings *durch die *Statistical Learning Theory *geliefert, sondern auch mit *Random-Forest*-Klassifizierern und *Bagging*-Methoden einige der heutzutage meist verwendeten *Machine-Learning*-Verfahren für Datenanalyse und Prädiktion entwickelt. Diese, wie auch Weiterentwicklungen klassischer statistischer Methoden, werden heutzutage in Gebieten wie der medizinischen Diagnostik, Business Analytics, Bildverarbeitung und in autonomen Systemen intensiv eingesetzt. Mit interpretierbaren Modellen, fundierten Ansätzen zur Quantifizierung von Unsicherheiten und Bewertung von Replizierbarkeit sowie substantiellen Fortschritten bei der statistischen Inferenz für Big-Data-Analysen tragen die moderne Statistik und Stochastik auch zu aktuellen Entwicklungen und Forschungstrends entscheidend bei. Statistische Expertise ist ebenfalls an vielen Stellen relevant für verbesserte Algorithmen und deren Verständnis. So ist die statische Kreuzvalidierung ein wichtiges Instrument für die Trainingsphase von *Deep Learnern*, um eine gute Generalisierungsfähigkeit zu erreichen.

Die Spezialisierung der Statistik als Disziplin auf einzelne Wissenschaftsfelder und der Einbezug von Expertenwissen über diese Felder hat zur Etablierung von Teildisziplinen wie der *Biometrie/Biostatistik, Umweltstatistik, Industriestatistik *oder der *Ökonometrie *geführt. Die technologischen Entwicklungen in der Rechentechnik und die Digitalisierung von Gesellschaft, Wirtschaft und empirischen Wissenschaften wurden in der Statistik frühzeitig aufgegriffen und haben viele Teilgebiete nachhaltig verändert. Insbesondere entstand die neue Teildisziplin der *Rechnergestützten Statistik*. Ebenso haben sich *Hochdimensionale Statistik *und *Statistisches Maschinelles Lernen *als Forschungsgebiete der Statistik etabliert und in Form eines erweiterten Methodenspektrums Eingang in anwendungsbezogene Gebiete (insbes. *Ökonometrie, Empirische Wirtschaftsforschung, Biostatistik, Technische Statistik*) gefunden. Die neuen Möglichkeiten durch Hardware und Software erlauben ebenfalls deutlich komplexere stochastische Modellierungen und Methoden zur Etablierung und Anwendung der benötigten statistischen Theorien.

Viele der unter dem Begriff *Data Science *adressierten Themen und Probleme finden in diesen Entwicklungen der Statistik ihre natürlichen Anknüpfungspunkte und wissenschaftliche Zitationsbasis, auch wenn sie durchaus neue Herausforderungen formulieren. Dies sowohl für die disziplinäre Forschung in der *Mathematischen *und *Angewandten Statistik *und *Stochastik*, als auch für interdisziplinäre Forschung und Datenanalyse. Hier sind insbesondere die Ökonometrie, Industriestatistik, Ausbildung und Lehre und die Amtliche Statistik zu nennen. Es ist festzustellen, dass eigenständige Initiativen zu *Data Science *oder *Data Literacy *besonders in denjenigen Wissenschaftsfeldern entstehen, in denen der Siegeszug der Statistik im letzten Jahrhundert nicht nachhaltig Eingang gefunden hat in Form der Etablierung von Statistikprofessuren oder der Herausbildung einer selbstständigen statistischen Teildisziplin.

Die valide Analyse von sehr großen Datenbeständen erfordert substantielle Beiträge der Statistik und somit qualifizierte Statistiker, die umfassende Kompetenzen auch in Bereichen wie *Machine Learning, Data Privacy and Literacy, paralleles Rechnen, Algorithmik *und *Optimierung *besitzen. Eine rein algorithmische Sichtweise, die *Data Science *als Ingenieursfach oder Teilgebiet der Informatik versteht und auf eine datenverarbeitende, algorithmische Sicht reduziert, greift deutlich zu kurz und wird selbst gesteckte Ziele wie Unsicherheitsquantifizierung oder Erklärbarkeit von KI nicht erreichen können. Sie läuft insbesondere auch Gefahr, die grundsätzliche wissenschaftstheoretische Erkenntnis zu ignorieren, dass eine Ergebnisinterpretation und die Bewertung von Datenunsicherheit Statistik benötigt in Form von wissenschaftlichen, falsifizierbaren Modellen, die Mechanismen der Datengenerierung berücksichtigen.

Statistik zeigt die Möglichkeiten und Grenzen der Wissensextraktion aus Daten auf und stellt somit auch die Grundlage für einen kritischen Umgang mit Daten dar. Statistik war und ist die Wissenschaft für die Erkenntnisgewinnung aus Daten und jegliche Datenwissenschaft ist ohne Statistik nicht denkbar.

Vor dem Hintergrund einer Analyse in jüngerer Zeit national und international aufgelegter Programme und etablierter Studiengänge sowie neu geschaffener Forschungsstrukturen an renommierten Einrichtungen spricht die Deutsche Statistische Gesellschaft die folgenden Empfehlungen und Forderungen aus.

Positionen und Empfehlungen der Deutschen Statistischen Gesellschaft im Einzelnen:

- Als Fachgesellschaft für theoretische, angewandte und praktische Statistik, die auch Statistikerinnen und Statistiker mit Expertenwissen aus Anwendungsgebieten vertritt, versteht die Deutsche Statistische Gesellschaft Data Science und Data Literacy als integrale Teilgebiete.
- Die Deutsche Statistische Gesellschaft befürwortet und fordert den Ausbau der Statistik an Hochschulen durch Einrichtung neuer Professuren und Stellen für wissenschaftliche Mitarbeiter/innen, um dem substantiell gestiegenen Bedarf an Kompetenzen und Fähigkeiten im Bereich Statistik, Digitalisierung, Data Literacy und Data Science in Lehre und Forschung nachzukommen. Dies ist insbesondere notwendig, damit sich der substantiell wachsende Lehr- und Schulungsbedarf nicht zu Lasten der Forschungsqualität auswirkt. Ein Stellenaufwuchs ist insbesondere in Hinblick auf wirtschafts- und ingenieurwissenschaftliche Fakultäten und Studiengänge notwendig, um der gestiegenen Bedeutung der Thematik für den Wirtschaftsstandort Deutschland gerecht zu werden.
- Die statistische Beratung und nicht-curriculare statistische Schulungsangebote an Hochschulen müssen bedarfsgerecht ausgebaut werden.
- An Lehr- und Forschungsstrukturen zu *Data Science, Machine Learning, KI *und *Data Literacy *(z. B. Förderprogramme, Studiengänge, Doktorandenprogramme, Forschungsverbünde, Forschungsprogramme) sowie digitalisierte Lehr- und Ausbildungsangebote (E-Learning) müssen in Statistik qualifiziert ausgebildete Lehrende und Forschende beteiligt werden. Sie sollten in die Leitung und Koordinierung eingebunden sein.
- Der Aufbau von Strukturen in Lehre und Forschung ohne Verbindung zu bestehenden Statistikinstituten bzw. -professuren und ohne maßgebliche statistische Fachkompetenz wird den Anforderungen nicht gerecht. Die Entwicklung und Vermittlung von Datenanalyse-Kompetenzen gehört in die Hände von qualifizierten Statistiker/innen. Die Deutsche Statistische Gesellschaft empfiehlt hier dringend, dies in der weiteren Entwicklung zu berücksichtigen und ggfs. nachzubessern.
- Standardisierte, breitenorientierte (etwa fächerübergreifende) Online-Learning Angebote zu Themen wie *Data Science *und *Data Literacy *sind im Sinne eines ergänzenden Angebots zu begrüßen. Sie dürfen Lehrende und die Vielfalt der Lehre und Didaktik jedoch nicht einschränken, sondern sollten diese bereichern, und können fachspezifische Lehre nicht ersetzen

#### Die Rolle der Statistik in der Künstlichen Intelligenz

*DAGStat (März* *2020)*

##### Zusammenfassung und Diskussion

Die Statistik ist eine breit angelegte wissenschaftsübergreifende Fachrichtung. Mit ihrem Spezialwissen über Datenauswertungen angefangen von der Fragestellung über Design und Analyse bis hin zur Interpretation hat sie eine wichtige und besondere Rolle. Als Kernelement der KI ist sie der natürliche Partner für andere Disziplinen in Lehre, Forschung und Praxis. Insbesondere lassen sich folgende Beiträge der Statistik für die künstliche Intelligenz zusammenfassen:

1. Methodische Entwicklung: Die Entwicklung von KI-Systemen und ihre theoretische Unterfütterung hat sehr stark von Forschungen in den Computerwissenschaften und der Statistik profitiert und so manches Verfahren wurde von Statistikern entwickelt. Neuere Entwicklungen wie Extreme Learning Machines zeigen, dass die Statistik auch für die Konzeption von KI-Systemen wichtige Beiträge liefert, z. B. durch verbesserte Lernalgorithmen basierend auf penalisierten oder robustifizierten Schätzverfahren.
2. Planung und Design: Die Statistik kann dazu beitragen, die Datenerhebung bzw. Aufbereitung (Fallzahlen, Sampling Design, Gewichtung, Einschränkung des Datensatzes, Design of Experiments, etc.) für die anschließende Auswertung mit KI-Methoden zu optimieren. Außerdem können die Gütemaße der Statistik und ihre zugehörigen Inferenzmethoden bei der Bewertung von KI-Modellen helfen.
3. Beurteilung von Datenqualität und Datenerhebung: Die explorative Datenanalyse stellt ein breites Spektrum an Werkzeugen zur Verfügung, die empirischen Verteilungen der Daten zu visualisieren und entsprechende Kennzahlen abzuleiten, die sowohl dazu genutzt werden können Anomalien festzustellen oder Bereiche typischer Werte festzulegen, um Eingabefehler zu korrigieren, Normwerte zu bestimmen und fehlende Werte zu imputieren. Im Zusammenspiel mit Standardisierungen in der Datenablage können hier Datenfehler im Messprozess frühzeitig erkannt und korrigiert werden. Mit Hilfe modellbasierter statistischer Methoden ist außerdem ein umfassendes Parametertuning auch für kleines Datenaufkommen möglich.
4. Unterscheidung von Kausalität und Assoziationen: In der Statistik sind Methoden zum Umgang mit Drittvariableneffekten bekannt. Hier gilt es, theoretisch informiert zu differenzieren, welches Verhältnis in den Beobachtungsdaten vorhandene Drittvariablen zu Treatment und Outcome haben, um Bias in den Schätzern kausaler Effekte zu vermeiden. Pearls kausales Framework ermöglicht dabei die Analyse kausaler Effekte sowie die Simulation von Interventionen. Die Integration kausaler Methoden in die KI trägt auch dazu bei, die Transparenz und Akzeptanz von KI-Methoden zu stärken.
5. Einschätzung von Sicherheit bzw. Unsicherheit in Ergebnissen: Die Statistik kann dazu beitragen, die Quantifizierung von Unsicherheit und die Interpretierbarkeit von KI Methoden zu ermöglichen oder zu verbessern. Durch Annahme spezifischer stochastischer Modelle können außerdem mathematische Validitätsbeweise geliefert werden. Zudem werden durch die Bereitstellung stochastischer Simulationsdesigns Grenzen der Methoden ausgelotet.
6. Eine gewissenhafte Umsetzung der Punkte 2–5 inklusive vorher festgelegtem Auswertungsplan wirkt zudem der Replikationskrise in vielen Wissenschaftsbereichen entgegen. In dieser seit Beginn der 2010er Jahre andauernden methodischen Krise hat sich herausgestellt, dass viele Studien insbesondere in der Medizin und den Sozialwissenschaften nur schwer oder gar nicht reproduzierbar sind.
7. Bildung, Weiterbildung und Öffentlichkeitsarbeit: Mit ihrem Spezialwissen ist die Statistik der natürliche Partner für andere Disziplinen in Lehre und Weiterbildung. Gerade bei der Weiterentwicklung der Methoden der Künstlichen Intelligenz kann die Statistik den wissenschaftlichen Austausch stärken.

Dabei unterstützt die DAGStat ohne Einschränkung die von der Datenethikkommission der Bundesregierung im Oktober 2019 veröffentlichten Grundsätze. Künstliche Intelligenz muss sich in all ihren Anwendungen ethisch, rechtlich, kulturell und institutionell in die Gesellschaft einbetten. Dies dient dazu, eine verantwortungsvolle und gemeinwohlorientierte Entwicklung und Nutzung zu erreichen. Die DAGStat setzt sich auch dafür ein, dass ein für alle Disziplinen und Anwender verbindlicher Ordnungsrahmen national wie international festgelegt wird und bietet an, diesen fachspezifisch unter Einbringung von Expertenwissen mitzugestalten.

## Footnotes

1 Allerdings findet Frohn (2004) keine empirischen Belege für diese Vermutung.

2 Das Motto war noch zur Amtszeit des amerikanischen Präsidenten Donald Trump festgelegt worden. Allerdings gab es auch Kommentare, wonach die Statistik vielleicht großartig aber noch nie groß war.

3 Das Kolloquium fand zu einem Zeitpunkt statt, an dem das Wort COVID 19 noch nicht erfunden worden war. Deswegen findet hier die Corona-Pandemie keine Erwähnung. Doch es ist nicht so, dass die Vertreter der Statistik zum Thema Corona geschwiegen hätten. Erwähnt seien hier exemplarisch die monatlichen Berichte der COVID-19 Data Analysis Group am Institut für Statistik der LMU unter https://www.covid19.statistik.uni-muenchen.de/newsletter/index.html oder auch Rendtel et al. (2021a) über die räumlich-zeitliche Darstellung von Corona-Inzidenzen.

4 In vielen Fällen handelt es sich bei „Erhebungen“ jedoch nur um Auswertungen administrativer Datenquellen.

5 Traditionell ist der Präsident des Statistischen Bundesamts ein Jurist und der Vizepräsident hat einen Abschluss in VWL. Formell festgeschrieben ist dies jedoch nicht.

6 Mittlerweile wurde der RatSWD (https://www.ratswd.de/) in eine noch umfangreichere Infrastruktur, das KonsortSWD (https://www.konsortswd.de/) eingebettet. Dieses Konsortium baut die Dateninfrastruktur im Bereich der Sozial‑, Verhaltens‑, Bildungs- und Wirtschaftswissenschaften aus. Es ist Teil einer von der DFG geförderten National Forschungsdaten Infrastruktur (https://www.dfg.de/foerderung/programme/nfdi/).

7 In Berlin wurde das Programm in einen allgemeinen Master Studiengang Statistik eingegliedert.

8 https://ec.europa.eu/eurostat/de/web/european-statistical-system/emos (Aufgerufen am 06.10.2020).

9 Den EMOS Wettbewerb (https://ec.europa.eu/eurostat/cros/content/ntts-2019-emos-master-thesis-competition_en aufgerufen 06.10 2020) von Eurostat und den Gerhard-Fürst-Preis des Statistischen Bundesamts, siehe Erfurth (2020) sowie https://www.destatis.de/DE/Ueber-uns/Unsere-Aufgaben/Gerhard-Fuerst-Preis/preistraeger.html für weitere Fürst-Preis Gewinner.

10 https://www.elitenetzwerk.bayern.de/start/foerderangebote/elitestudiengaenge.

11 Siehe http://www.stat.fu-berlin.de/. Neben individuellen Beratungen bei Master- und Doktorarbeiten bietet fu:stat auch Kurse zu speziellen Themen an. Neu ist ein Wiki mit Anleitungen für die Erstellung empirischer Auswertungen, vgl. http://www.stat.fu-berlin.de/wiki/index.html. Weiterhin werden auch Beratungen außerhalb der FU durchgeführt.

12 Siehe https://www.stablab.stat.uni-muenchen.de/index.html.

13 Allerdings wurde den Statistik-Empfehlungen in der Corona-Krise nicht immer gefolgt, vgl. zum Beispiel Rendtel et al. (2021b).

14 Zur Entwicklung der amtlichen Statistik siehe Radermacher (2020, insbesondere Kapitel 2 ‚Official Statistics – Public Informational Infrastructure‘, S. 11–49).

15 BVerfG, Urteil v. 15. Dezember 1983, Az. 1 BvR 209, 269, 362, 420, 440, 484/83.

16 Siehe hierzu u. v. a. BfDI (2009).

17 Orwell (1949).

18 Hierzu auch Grohmann (2011).

19 Hierzu Müller et al. (1991).

20 Hierzu Statistisches Bundesamt (1999).

21 Kommission zur Verbesserung der informationellen Infrastruktur zwischen Wissenschaft und und Statistik (2001).

22 Zu den damaligen Diskussionen und insbesondere zum Ausbau der informationellen Infrastruktur bis 2008 siehe die gemeinsame Festschrift für Johann Hahlen zum 65. Geburtstag und Hans Jürgen Krupp zum 75. Geburtstag, Rolf et al. (2008).

23 (Download 18. Oktober 2021).

24 Z. B. http://www.forschungsdatenzentrum.de/de/verarbeitendes-gewerbe/afid-panel-industriebetriebe (Download 18. Oktober 2021).

25 Siehe hierzu auch die Ausführungen von Rendtel und Wagner in diesem Aufsatz.

26 Geändert durch Verordnung (EU) 2015/759 des Europäischen Parlaments und des Rates vom 29. April 2015; https://eur-lex.europa.eu/legal-content/DE/TXT/PDF/?uri=celex:32015R0759 (Download 18. Oktober 2021).

27 https://ec.europa.eu/eurostat/de/web/quality/european-quality-standards/european-statistics-code-of-practice (Download 18. Oktober 2021).

28 Zur deutschen amtlichen Statistiktradition siehe insbesondere auch Desroisieres (2005).

29 Siehe hierzu https://www.ilo.org (Download 18. Oktober 2021).

30 Hierzu z. B. Litz (2000).

31 ‚The place where a person normally spends the daily period of rest, regardless of temporary absences for purposes of recreation, holidays, visits to friends and relatives, business, medical treatment or religious pilgrimage.‘ siehe https://ec.europa.eu/eurostat/cache/metadata/en/demo_pop_esms.htm. (Download 18. Oktober 2021).

32 Zum Adäquationsprinzip siehe Grohmann (1985).

33 Zur Frankfurter Schule in der Statistik siehe auch von der Lippe (2013), der in diesen Ausführungen die Frankfurter Schule kritisiert aber aus heutiger Sicht dieser durchaus zugeordnet werden kann.

34 https://digital-strategy.ec.europa.eu/en/news/experts-say-privately-held-data-available-european-union-should-be-used-better-and-more (Download 18. Oktober 2021).

35 Zur Nutzung von Mobilfunkdaten als experimentelle Daten (EXDAT) in der amtlichen Statistik siehe https://www.destatis.de/DE/Service/EXDAT/_inhalt.html#sprg415070 (Download 18. Oktober 2021), zu EXDAT siehe Hauf et al. (2020).

36 https://www.cbs.nl/nl-nl/uitgelicht/statement-pilot-t-mobile-en-cbs (Download 18. Oktober 2021).

37 Zur Datenqualität im ESS siehe https://ec.europa.eu/eurostat/de/web/products-manuals-and-guidelines/-/ks-gq-19-006 (Download 18. Oktober 2021).

38 https://ec.europa.eu/eurostat/cros/news/scheveningen-memorandum-big-data-and-official-statistics-adopted-essc_en (Download 18. Oktober 2021).

39 Zu den Projekten im Europäischen Statistischen System Big Data I und II siehe https://ec.europa.eu/eurostat/cros/content/essnet-big-data-1_en (Download 18. Oktober 2021), zu den Entwicklungen in Deutschland u. a. Wiengarten und Zwick (2017).

40 DGINS Bucharest https://dgins2018.statisticsevents.ro/bucharest-memorandum/ (Download 18. Oktober 2021).

41 Das Projekt hat Anfang 2021 begonnen, eine eigene Webseite des Projektes wird derzeit noch nicht angeboten. Zur weiteren Information siehe 5th meeting of the Working Group on Methodology (WGM). Luxembourg, 1 April 2020, Item ‚Web Intelligence Hub‘, https://ec.europa.eu/eurostat/cros/content/11-web-intelligence-hub_en (Download 18. Oktober 2021).

42 Siehe hierzu vor allem auch Kühnemann (2021).

43 https://ec.europa.eu/eurostat/cros/content/essnet-smart-surveys_en (Download 18. Oktober 2021).

44 Siehe hierzu Blaudow (2019).

45 https://www.destatis.de/DE/Service/EXDAT/_inhalt.html (Download 18. Oktober 2021).

46 https://www.destatis.de/DE/Service/EXDAT/Datensaetze/mobilitaetsindikatoren-mobilfunkdaten.html (Download 18. Oktober 2021).

47 Siehe hierzu auch DAGStat (2021).

48 https://uksa.statisticsauthority.gov.uk/ (Download 18. Oktober 2021).

49 Vgl. die entsprechenden Analysen mit einer vergleichbaren früheren Umfrage in Wagner (2011).

50 Es sei hier daran erinnert, dass das BStatG seit 2016 in § 13a ausdrücklich vorsieht, dass die Deutsche Bundesbank Daten aus den von ihr erstellten Wirtschaftsstatistiken an das Statistische Bundesamt übermitteln darf. Meines Wissens wird davon allerdings bisher kein Gebrauch gemacht.

51 Mir ist zumindest kein einziger entsprechender Beitrag bekannt.

52 „Laßt, die ihr eingeht, jede Hoffnung fahren“.

53 Vgl. für eine umfassende Darstellung der Entwicklung der Möglichkeiten von externen Wissenschaftlern für die Arbeit mit Firmendaten der amtlichen Statistik Wagner (2015).

54 Für Studierende und Promovierende gibt es Sonderregelungen mit vergünstigten Finanzierungsbedingungen, die dieses Problem abmildern; vgl. www.forschungsdatenzentrum.de.

55 Vgl. hierzu https://fdz.iab.de/de/FDZ_Data_Access/FDZ_Remote_Data_Access/faq.aspx.

56 Genauer gesagt, mit den jeweiligen Inhabern der dortigen Statistik-Lehrstühle, Ulli Rendtel und Ralf Münnich.

57 Der Arbeitsmarkt im Bereich der amtlichen Statistik ist eher überschaubar.

58 In einigen Fällen ging dem Angebot über eine Festanstellung ein Praktikum und/oder eine externe Masterarbeit voraus und manche dieser Fälle sind hier explizit erwähnt.

59 Dies war ein Kritikpunkt bei Einrichtung des Studiengangs.

60 Einzige Auflage: Explizites Erwähnen von *quantitativen* Methodenkenntnissen im Umfang von 30 ECTS-Punkten als Zulassungskriterium.

61 Eine S‑Professur ist an die Tätigkeit an einer außeruniversitären Forschungseinrichtung gekoppelt. Gleichzeitig werden an der Universität Promotionen betreut und in verringertem Umfang Lehrveranstaltungen angeboten.

62 Viel gebräuchlicher ist jedoch der modische Ausdruck „Machine Learning“.

63 https://gi.de/fileadmin/GI/Allgemein/PDF/GI_Arbeitspapier_Data-Science_2019-12_01.pdf.

64 Zum KI-Programm der Bundesregierung, siehe https://www.bmbf.de/de/kuenstliche-intelligenz-5965.html (Abgerufen am 06.10.2020).

65 Zwar fordert die Bundesregierung in ihrer aktuellen Datenstrategie die Einrichtung von Datennutzungsbeauftragten (Chief Data Scientists) in allen Bundesministerien, die zusammen mit den Datenschutzbeauftragten für eine effiziente Datennutzung sorgen sollen, siehe https://www.bundesregierung.de/breg-de/suche/datenstrategie-der-bundesregierung-1845632 (Abruf 06.10.2021). Dies ist aber nur ein schwacher Trost. Die Einrichtung von Statistik-Professuren besitzt eine deutlich größere öffentliche Wirkung.

66 https://www.fu-berlin.de/service/zuvdocs/amtsblatt/2019/ab152019.pdf.

67 Neben der Erwähnung der Statistikfelder in der obigen Übersicht wurde in dem Dokument durchgängig Mathematik durch Mathematik/Statistik ersetzt.

68 Die Originalfassung findet man unter https://dstatg.de/positionspapier-die-rolle-der-statistik-fuer-big-data-data-literacy-machine-learning-ki-analytics-und-data-science.

69 https://www.dagstat.de/fileadmin/dagstat/documents/DAGStat_KI_Stellungnahme_200303.pdf.
